# Postgraduate ethics training programs: a systematic scoping review

**DOI:** 10.1186/s12909-021-02644-5

**Published:** 2021-06-09

**Authors:** Daniel Zhihao Hong, Jia Ling Goh, Zhi Yang Ong, Jacquelin Jia Qi Ting, Mun Kit Wong, Jiaxuan Wu, Xiu Hui Tan, Rachelle Qi En Toh, Christine Li Ling Chiang, Caleb Wei Hao Ng, Jared Chuan Kai Ng, Yun Ting Ong, Clarissa Wei Shuen Cheong, Kuang Teck Tay, Laura Hui Shuen Tan, Gillian Li Gek Phua, Warren Fong, Limin Wijaya, Shirlyn Hui Shan Neo, Alexia Sze Inn Lee, Min Chiam, Annelissa Mien Chew Chin, Lalit Kumar Radha Krishna

**Affiliations:** 1 Yong Loo Lin School of Medicine, National University of Singapore, NUHS Tower Block, 1E Kent Ridge Road, Level 11, Singapore, 119228 Singapore; 2 Division of Supportive and Palliative Care, National Cancer Centre Singapore, 11 Hospital Cr, Singapore, 169610 Singapore; 3 Duke-NUS Medical School, National University of Singapore, 8 College Rd, Singapore, 169857 Singapore; 4 Department of Rheumatology and Immunology, Singapore General Hospital, 16 College Road, Block 6 Level 9, Singapore General Hospital, Singapore, 169854 Singapore; 5 Department of Infectious Diseases, Singapore General Hospital, Outram Road, Singapore, 169608 Singapore; 6 Division of Cancer Education, National Cancer Centre Singapore, 11 Hospital Cr, Singapore, 169610 Singapore; 7 Medical Library, National University of Singapore Libraries, Blk MD6, Centre, 14 Medical Dr, #05-01 for Translational Medicine, Singapore, 117599 Singapore; 8 Palliative Care Institute Liverpool, Cancer Research Centre, University of Liverpool, 200 London Rd, L3 9TA Liverpool, UK; 9 Centre of Biomedical Ethics, National University of Singapore, 21 Lower Kent Ridge Rd, Singapore, 119077 Singapore; 10 PalC, The Palliative Care Centre for Excellence in Research and Education, PalC c/o Dover Park Hospice, 10 Jalan Tan Tock Seng, Singapore, 308436 Singapore

**Keywords:** Postgraduate medical education, Physicians, Medical ethics, Ethics training program, Ethics education, Ethics curriculum, Scoping review, Systematic scoping review, SEBA

## Abstract

**Background:**

Molding competent clinicians capable of applying ethics principles in their practice is a challenging task, compounded by wide variations in the teaching and assessment of ethics in the postgraduate setting. Despite these differences, ethics training programs should recognise that the transition from medical students to healthcare professionals entails a longitudinal process where ethics knowledge, skills and identity continue to build and deepen over time with clinical exposure.

A systematic scoping review is proposed to analyse current postgraduate medical ethics training and assessment programs in peer-reviewed literature to guide the development of a local physician training curriculum.

**Methods:**

With a constructivist perspective and relativist lens, this systematic scoping review on postgraduate medical ethics training and assessment will adopt the Systematic Evidence Based Approach (SEBA) to create a transparent and reproducible review.

**Results:**

The first search involving the teaching of ethics yielded 7669 abstracts with 573 full text articles evaluated and 66 articles included. The second search involving the assessment of ethics identified 9919 abstracts with 333 full text articles reviewed and 29 articles included. The themes identified from the two searches were the goals and objectives, content, pedagogy, enabling and limiting factors of teaching ethics and assessment modalities used. Despite inherent disparities in ethics training programs, they provide a platform for learners to apply knowledge, translating it to skill and eventually becoming part of the identity of the learner. Illustrating the longitudinal nature of ethics training, the spiral curriculum seamlessly integrates and fortifies prevailing ethical knowledge acquired in medical school with the layering of new specialty, clinical and research specific content in professional practice. Various assessment methods are employed with special mention of portfolios as a longitudinal assessment modality that showcase the impact of ethics training on the development of professional identity formation (PIF).

**Conclusions:**

Our systematic scoping review has elicited key learning points in the teaching and assessment of ethics in the postgraduate setting. However, more research needs to be done on establishing Entrustable Professional Activities (EPA)s in ethics, with further exploration of the use of portfolios and key factors influencing its design, implementation and assessment of PIF and micro-credentialling in ethics practice.

**Supplementary Information:**

The online version contains supplementary material available at 10.1186/s12909-021-02644-5.

## Introduction

Seen as a means of ensuring that “obligations of moral nature which govern the practice of medicine” [1] are maintained, ethics training amongst physicians have evolved to contend with ethical issues facing medical practice. Whilst basic levels of ethics knowledge and skills have been stipulated by accreditation bodies such as The Royal College of Physicians and Surgeons of Canada, The General Medical Council, the American Academy of Family Physicians (AAFP) and the Accreditation Council for Graduate Medical Education (ACGME), many ethics programs have struggled to keep pace with change whilst remaining sensitive to the demands of clinical practice. Inevitable variations in the content and duration of ethics education amongst physicians have been laid bare in a recent review pertaining to family physicians in residency programs in the United States [2].

The litmus test for effectively educating physicians in ethics knowledge, skills and professional conduct in a medical field trepidatious of legal recourse and struggling to meet public trust and societal expectations [3–7] has perhaps been the COVID-19 pandemic. Yet, the surfacing of reports of questionable physician conduct and clinical decisions during the COVID-19 pandemic also offers an opportunity to take stock of prevailing education programs, review gaps in content and structure of ethics education programs as well as update and instil more evidence based, clinically relevant, learner centred education initiatives.

### The need for this review

To guide this process of retooling ethics education programs for physicians, a systematic scoping review is proposed to analyse current postgraduate medical ethics training and assessment programs in peer-reviewed literature.

## Methodology

We adopt Krishna’s systematic evidence-based approach (SEBA) to guide this systematic scoping review (henceforth SSRs in SEBA) [8–14] and scrutinise a broad range of literature [15–17]. With its constructivist perspective and relativist lens, SSRs in SEBA map the complex and diverse historical, socio-cultural, ideological and contextual factors that impact practice to provide a holistic picture of medical ethics training programs for graduates beyond medical school [17–24].

**Fig. 1:**
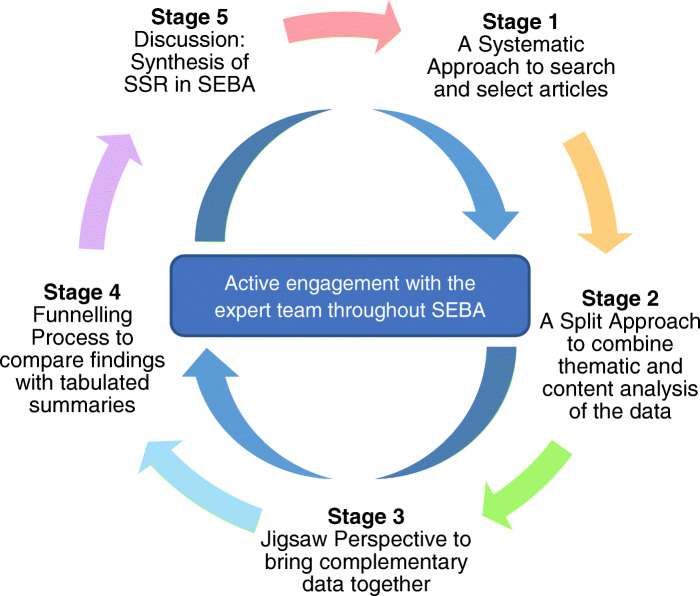
The SEBA process

To further improve the reliability of the results, the research team consulted medical librarians from the Yong Loo Lin School of Medicine (YLLSoM) at the National University of Singapore (NUS) and the National Cancer Centre Singapore (NCCS), and local educational experts and clinicians at NCCS, Palliative Care Institute Liverpool, YLLSoM and Duke-NUS Medical School (henceforth the expert team). The Systematic Approach, Split Approach, Jigsaw Perspective, , Funnelling Process, and Discussion stages of SEBA (Fig. 1. The SEBA Process) were used to guide the entire research process.

### Stage 1: Systematic approach

#### Determining the title and background of the review

The research team consulted the expert team and stakeholders from a local medical ethics training program to determine the overarching goals of the SSR in SEBA as well as the population, context and medical ethics training programs to be evaluated.

#### Identifying the research question

Guided by the Population, Intervention, Comparison and Outcome (PICOS) elements of the inclusion criteria [25], the primary research question is “*How do postgraduate medical training programs teach ethical skills*?” The secondary questions are “*What are the core topics included*?” and “*What are the methods used to structure the program in postgraduate training*?”

As part of the SEBA methodology’s iterative process, when the initial results of this review were discussed, the expert team advised that a study of current methods of assessing ethics be conducted to address the lack of data on assessments of ethics education. Thus, a second SSR in SEBA was carried out. Similarly guided by PICOS, the primary research question is *“How is ethics knowledge, skills, and competencies assessed in postgraduate training?”* The secondary question is *“What domains are assessed?”*

#### Inclusion criteria

Guided by the expert team, the research team created the inclusion criteria for the SSRs in SEBA for teaching and assessing medical ethics, as outlined (Table 1).

**Table 1:** PICOS, inclusion criteria and exclusion criteria applied to literature search on medical ethics training programs

**Teaching of ethics**		
**PICOS**	**Inclusion criteria**	**Exclusion criteria**
Population	Junior doctors, residents, senior residents, registrars and or medical officers undergoing postgraduate training	Undergraduate and postgraduate medical students Allied health specialties such as Pharmacy, Dietetics, Chiropractic, Midwifery, Podiatry, Speech Therapy, Occupational and Physiotherapy Non-medical specialties such as Clinical and Translational Science, Alternative and Traditional Medicine, Veterinary, Dentistry
Intervention	Practices in nurturing and teaching ethics of doctors	
Comparison	Comparisons of the various practices (approaches, modalities, processes, objectives, motivations, challenges, facilitating characteristics/resources)	
Outcome	Approaches, modalities, processes, objectives, motivations, challenges, facilitating characteristics/resources in nurturing and teaching ethics Impact of teaching ethics on host organisation, assessors, and assessments	
Study Design	Articles in English or translated to English All study designs including: o Mixed methods research, meta-analyses, systematic reviews, randomised controlled trials, cohort studies, case-control studies, cross-sectional studies, and descriptive papers Year of Publication: 1 January 1990–31 December 2019 Databases: PubMed, Embase, PsycINFO, ERIC	Grey Literature / electronic and print information not controlled by commercial publishing Articles focusing on non-human subjects
**Assessing of ethics**		
Population	Junior doctors, residents, senior residents, registrars and or medical officers undergoing postgraduate training	Undergraduate and postgraduate medical students Allied health specialties such as Pharmacy, Dietetics, Chiropractic, Midwifery, Podiatry, Speech Therapy, Occupational and Physiotherapy Non-medical specialties such as Clinical and Translational Science, Alternative and Traditional Medicine, Veterinary, Dentistry
Intervention	Practices in assessing ethics of postgraduate doctors	
Comparison	Comparisons of the various practices (approaches, modalities, processes, objectives, motivations, challenges, facilitating characteristics/resources)	
Outcome	Approaches, modalities, processes, objectives, motivations, challenges, facilitating characteristics/resources in nurturing and teaching ethics Impact of teaching ethics on host organisation, assessors, and learners	
Study design	Articles in English or translated to English All study designs including: Mixed methods research, meta-analyses, systematic reviews, randomised controlled trials, cohort studies, case-control studies, cross-sectional studies, and descriptive papers Year of Publication: 1 January 1990–31 December 2019 Databases: PubMed, Embase, PsycINFO, ERIC	Grey Literature/electronic and print information not controlled by commercial publishing Articles focusing on non-human subjects

#### Searching

Overall, both searches involved 16 members of the research team who carried out independent searches of PubMed, Embase, PsycINFO, and ERIC databases for the review. In keeping with Pham, Rajic [26]’s approach to ensuring a viable and sustainable research process, the research team confined the searches to articles published between 1 January 1990 and 31 December 2019 to account for prevailing manpower and time constraints. All research methodologies in articles published in English or had English translations were included. The independent searches were carried out between 14 February 2020 and 9 April 2020. The full PubMed search strategy may be found in Additional File 1.

**Fig. 2:**
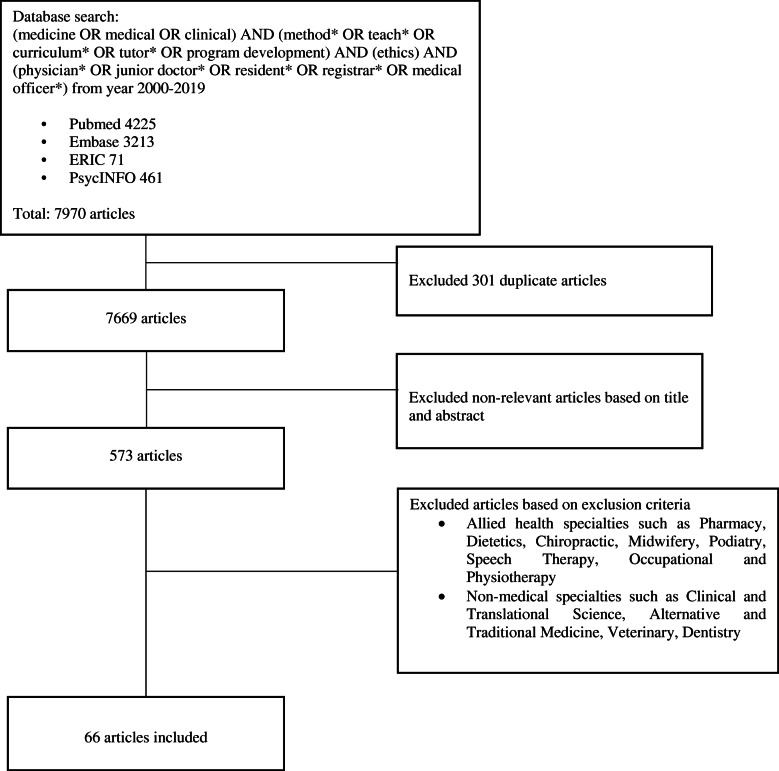
PRISMA Flow Chart for the Teaching of Ethics

**Fig. 3:**
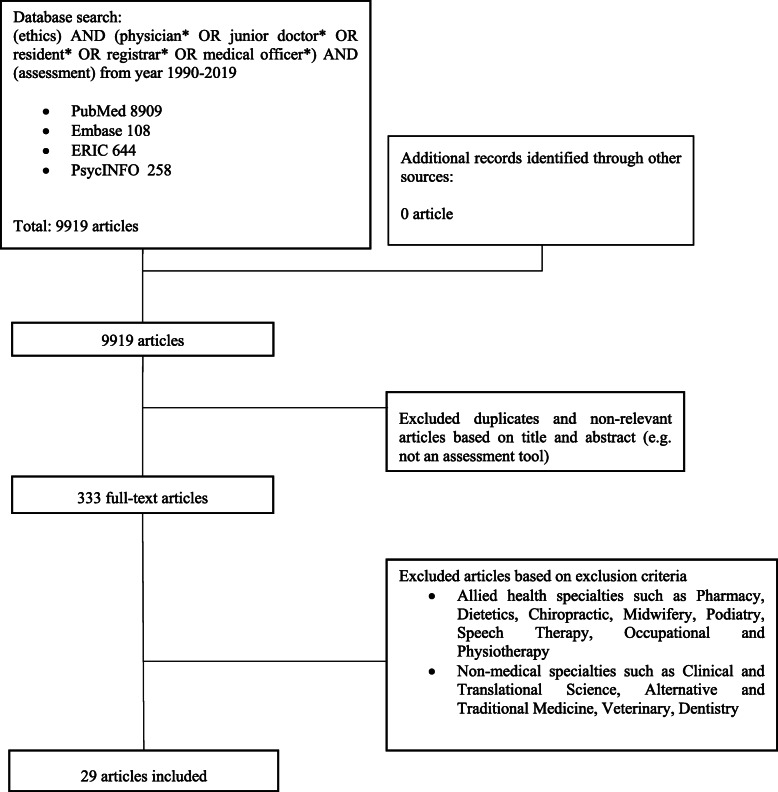
PRISMA Flow chart for the assessing of ethics

The research team then independently reviewed all the titles on the final list, compared their individual lists of articles to be included in the review and employed ‘negotiated consensual validation’ to achieve consensus on the final list of articles to be analysed on the teaching of ethics (Fig. 2.) and assessing of ethics (Fig. 3).

### Stage 2: Split approach 

For each SSR in SEBA, two teams of five researchers concurrently and independently reviewed the full-text articles in keeping with Krishna’s Split Approach that is focused on enhancing the reliability of the analyses [27, 28]. The first team scrutinised the included articles using Braun and Clarke [29]’s approach to thematic analysis whilst the second team employed Hsieh and Shannon [30]’s approach to directed content analysis. Comparisons between the results of the Split Approach provides method triangulation whilst having each reviewer independently analyse the same data provides investigator triangulation [27, 28]. Triangulation augments external validity and allows this approach to be more objective. 

#### Braun and Clarke (2006)’s approach to thematic analysis

Without an a priori framework for either teaching or assessing medical ethics amongst physicians, we employed Braun and Clarke’s approach to thematic analysis to single out common themes across varying goals and populations of physicians of different grades, experiences and specialties whilst circumnavigating the context-specific nature of medical ethics in Medicine [29, 31–37]. It also accommodates for a wide range of research methodologies present amongst the included articles which prevents the use of statistical pooling and analysis [29, 38–42] and facilitates appropriate analysis of socio-culturally influenced educational processes such as medical ethics.

‘Codes’ were constructed from the ‘surface’ meaning of the text through a reiterative step-by-step thematic analysis. These were re-organised into themes that were best able to represent the data. They were reviewed individually and then as a group. Subsequently, the members of this sub-team deliberated their separate findings online and utilised ‘negotiated consensual validation’ to achieve consensus on the final themes.

#### Hsieh and Shannon (2005)’s approach to directed content analysis

Hsieh and Shannon’s approach to directed content analysis was employed to increase the validity of the themes and to address Braun and Clarke’s relative failure to engage contradictory data.

 With regards to the teaching of ethics, the second sub-team drew codes and categories from Sutton [43]’s article entitled ‘*Ethics and law teaching and learning in undergraduate medicine*’ and McKneally and Singer [44]’s ‘*Bioethics for clinicians 25. Teaching bioethics in the clinical setting*’.

 With regards to the assessing of ethics, codes and categories from Norcini, Anderson [35]’s *‘Draft 2018 Consensus Framework for Good Assessment’*, Veloski, Boex [45]‘s ‘*Systematic review of the literature on assessment, feedback and physicians’ clinical performance: BEME Guide No. 7*′ and Watling and Ginsburg [46]’s *‘Assessment, feedback and the alchemy of learning’* were used*.*

These codes were adopted as a framework for reviewing the included articles. Any relevant data not captured by existing codes were assigned a new code through deductive category application. The independent findings were discussed online and ‘negotiated consensual validation’ was again used to achieve consensus on the final ‘code book’.

### Stage 3: The jigsaw perspective

The findings of the Split Approach and its reiterative process were then pooled together to ensure a well-rounded perspective of the data. Here, common themes and categories within each SSR were compared. Overlaps between the categories and themes were combined to create a wider perspective of the data, much like bringing together complementary pieces of a jigsaw. This process is called the Jigsaw Perspective and is overseen by the expert team to ensure consistency.

## Results

The first search involving the teaching of ethics retrieved 7669 abstracts, with 573 full-text articles reviewed and 66 articles included. Comparison of the categories and themes identified as part of the Split Approach revealed similar categories and themes which were combined into themes/categories using the Jigsaw Perspective. These themes/categories include the goals, content, teaching methods employed, and enablers and barriers to teaching ethics.

For the assessment of ethics, the search saw 9919 abstracts identified, 333 full-text articles reviewed and 29 articles included. The Split Approach from the SSR in SEBA of assessment methods revealed three themes/categories which included the types and domains assessed and the pros and cons of various assessment methods.

### Stage 4: The funnelling process

In addition, a third sub-team summarised and tabulated the included full-text articles to ensure that important concepts of discussion and contradictory views within the included articles were retained. The tabulated summaries also serve to verify that the results ascertained are an accurate representation of the existing data. The tabulated summaries for the teaching and assessing of ethics may be found in Additional File 2 and 3 respectively. Under the oversight of the expert team, the research team combined themes/categories from the two SSRs in SEBA based upon their similarities and their areas of overlap in keeping with the Funnelling Process.

The five funnelled themes/categories from the two searches are the goals and objectives, the content, pedagogy, enabling and limiting factors, and assessment tools.

#### Goals and objectives

The goals and objectives of ethics training programs for doctors are highlighted in Table 2 below.

**Table 2:** Goals and objectives of ethics training programs

Goal	Objective
**Build Knowledge**	To understand the historical background and definition of ethics [47, 48], social science, philosophy, religion and law and their relevance to clinical care [49–51].
	To gain knowledge and awareness of ethics issues relevant to individual practices in the course of patient care [47, 49, 51–58].
**Improve Skills**	Improve problem-solving skills by thinking critically and systematically when an ethical dilemma arises such as by providing opportunities for doctors to discuss ethical dilemmas [47–49, 51–53, 55, 57, 59–62]
	Appreciate the socio-cultural nuances and individual circumstances of the patient and/or their family in the context of the ethical dilemma [60, 62].
	Develop interpersonal skills to resolve ethical conflicts [48, 50, 55, 63–65].
	Reduce likelihood of physician making an ethical error or legal error [49, 50, 52].
	Overall, improve patient care and clinical decision making and adherence to ethical guidelines as part of research [50, 60, 66, 67].
**Change Attitudes and Professional Identity**	Develop appropriate attitudes, values that facilitate ethical conduct [68] [57, 58].
	Maintain high level of professionalism and ethical practice [49, 54].
	Increase self-awareness and understanding of professional boundaries [48, 49, 52].
	Prevent cynicism and detachment in patient interaction and gainincrease job satisfaction [48, 50, 52, 64].
	Help doctors become good teachers and future role models [69, 70].
**Fulfil Duty to Society**	Sustain and improve accountability to public [69, 70] to fulfil physicians’ ethical and service obligations [49, 60, 70,76].

Overall, the goal of most ethics programs was to refresh key ethical principles covered in medical schools [51], prepare physicians to tackle ethical dilemmas, and improve their confidence in doing so [59, 71, 77]. Some programs also introduced context and specialty-specific ethical dilemmas as highlighted in the next section on content covered [48, 53, 56, 70, 78–80].

#### Content covered

Content covered is outlined in Table 3.

**Table 3:** Domains of content covered in ethics training programs

Domains	Subdomains/Topics	References
***Basic Principles of Ethics***		
Ethical Theories and the Hippocratic Oath	–	[80]
Respect for Patient and Autonomy	Privacy and confidentiality Disclosure or non-disclosure to patients Informed consent Decision-making capacity and surrogate decision-making Informed refusal of medical interventions Informed consent in minors	[47, 49, 52, 53, 65, 78, 80–89]
Beneficence and Non-Maleficence	Medical failures and errors such as problems associated with the transfer of care Truth-telling	[49, 53, 58, 83, 87]
Justice	Access to healthcare Healthcare disparities Healthcare system Allocation of scarce resources	[53, 58, 60, 83–85, 89]
Care at End-of-Life	Patient advance directives Withholding and withdrawing life-sustaining interventions, medical futility Care for the dying, palliative versus curative care Determination of death	[59, 65, 73, 78, 81, 84, 86]
Communication Skills and Competencies	Patient communication such as breaking bad news, or communication of adverse outcomes Interprofessional communication Conflict resolution	[49, 54, 60, 65, 74, 75, 82, 85, 89–93]
Doctor-Patient Relationship	This may include understanding day-to-day interactions with patients and how one should conduct themselves professionally or may tackle specific circumstances such as the acceptance of gifts from patients. Doctors are also taught how to navigate conflicts of interest.	[49, 54, 58, 60, 80, 82, 84]
Ethics and Law	This may cover medicolegal issues such as with regards to expert witness testimony	[84, 92, 94]
Ethics and Philosophy	–	[61]
***More Specialised Content***		
Application of Ethics in consideration of Sociocultural Nuances and Particular Circumstances of Patients	This may involve being, in general, well equipped to tackle communication challenges due to cultural differences. It may also include family relationships of patients and employment status.	[58–60, 86]
Research Ethics	Publication ethics Ethical issues in human subject research or in research involving vertebral animals Good clinical practice in research The use of placebos	[48, 49, 54, 66, 70, 85]
With Regards to Medical Trainees, or being a Resident	Disclosure of trainee status Tension between education and best care for patients Hidden curriculum Moral distress	[49, 52, 60, 90]
Specialty-specific Ethical Dilemmas	Neonatal, perinatal and paediatric care “ethics of consent and [law] regarding minors with the legal authority to consent.” Surgery, cosmetic surgery such as how to take informed consent for surgical procedures Genetics Psychiatry, such as on psychiatry diagnoses, suicide, consultation liaison psychiatry Organ donation Dermatology such as “cultural and religious determinants of dermatologic health care” Infectious diseases such as treatment of highly contagious disease, vaccination and bioterrorism Obstetrics and gynaecology, such as adolescent sexuality, domestic violence and abuse, termination of pregnancy, maternal-fetal conflict, assisted reproduction and paternal rights	[49, 58, 59, 62, 65, 78, 83, 84, 95]
Interactions with Society at Large	With vendors With the pharmaceutical industry such as in issues of drug pricing With the media and advertising	[49, 54, 80, 84, 85, 91]
Relationship with Healthcare Institute	Negotiation of contract Whistle blowing	[49, 54]

Most training programs covered a varying number of topics.

Whilst Carrese, Malek [96] noted an overlap in the range of topics covered in ethics training for doctors and those for medical students, the authors explain that “educational materials offered to residents can typically be more complex and contextual than those intended for medical students, and ethical issues can be more nuanced and discussed in greater depth”.

#### Pedagogy

The diverse pedagogies are highlighted in Table 4 below.

**Table 4:** Pedagogy employed

Domains	Elaboration	References
Case-based approach	Case-based approaches may be integrated into many of the approaches below. An example of how a case-based approach may be utilised is through videotaped consultation or significant event analysis as presented by Chandra et al. (2017 and Oljeski et al. (2004). Sim et al. (2015) and Goodrich, Irvine, and Boccher-Lattimore (2005) interestingly used narratives in their teaching to showcase the human element at the centre of ethical dilemmas. Roberts et al. (1996) in describing their work on ethics teaching in psychiatry, mention a six step approach to ethical cases, from defining the case to creating context for reflection and review.	[48, 50, 51, 56, 57, 59, 67, 73, 81, 82, 86, 97, 98]
Online ethics modules	These may be made available for interested learners to utilisel in their own free time. However, Jain et al. (2011) highlight that the “value of web-based approaches warrants further investigation”.	[50, 85, 89]
Lectures and Seminar Sessions which may be termed as “Grand Rounds”	Such methods are more didactic, with key speakers who might be experts in the field sharing information on ethics principles.	[65–67, 78, 81, 88, 92, 94]
Group Discussions	Such as on key ethical issues or cases, and may serve as a platform for learners to voice their opinions, values and uncertainties. There might be a faculty leader present to guide discussion.	[49, 50, 58, 59, 65, 73, 86, 94, 99]
Research Opportunities	In these, students are given the opportunity to carry out research projects.	[100]
Hands-on Practice	Doctors may be asked to apply their ethical knowledge and practice demonstrating ethical competencies through the use of: · Simulation · Role Play · Practice with Standardized Patients A case-based approach may be used in conjunction with hands-on practice.	[58, 69, 74, 78, 86, 99]
Reflective Practice	This may be achieved through: · Writing, editing and publishing deliberation on ethical issues · Writing and reading poetry and pieces of written work related to doctors and patients	[56, 69, 99]
Observation and Shadowing	Learners may be invited to family meetings, ethics consultation and inpatient rounds where they observe a careful consideration of ethics being integrated into clinical decision-making.	[72, 99]
Role-modelling	Jain et al. (2011)’s survey on ethics teaching on psychiatry residents elucidated that the teaching was more memorable if learners were treated ethically by their teachers.	[50, 70, 97]
Bedside teaching	These are tutorials carried out by tutors by the bedside.	[69, 70]
Master Programs in Medical Ethics or Fellowships	These are formal certification programmes in the field of Medical Ethics.	[62, 70]
Educational Portfolios	Portfolios may be utilised in conjunction with mentorship in order to improve self-reflection.	[63, 69]
Mentoring Programs	These mentoring programs may be informal or formal.	[63, 69, 94]

There is great variation in the timing and duration of such training sessions. Formal teaching run by the host organisation or institution tended to come in the form of mandatory training programmes [80, 81] that span the course of a few years [62, 82] or  a single day [67]. Some programs are held over a few hours each year [58, 94], or each month or every few months as part of a wider residency training program [49, 59, 83].

Informal programs tended to be situated in more informal settings where refreshments are served and hierarchies are minimised [49, 59].

Different training programs utilised a combination of approaches to meet their objectives [82]. At the University of Toronto, Howard, McKneally [70] describes integrating formal bioethics teaching with “role modelling of ethical behaviour and bedside teaching around ethical issues”. The impact of this combination is echoed by Lang, Smith [97]’s survey of paediatric programme directors on how ethics is taught. Carrese, Malek [96]’s literature review of medical ethics training similarly highlighted the synergistic nature of the formal, informal and hidden curricula [77].

Other authors have proffered the use of a multidisciplinary approach to illustrate the intricacies of team based working in the healthcare setting [59, 69, 73, 101, 102].

#### Enabling factors and barriers

Enabling factors and barriers to the successful execution of ethics training programs may present themselves as follows (Table 5):

**Table 5:** Enabling factors and barriers to ethics training programs

**Enabling factors**	**Elaboration**	**References**
*Learning Environment*		
Safe environment	A non-judgemental, safe space inspires reflection, sharing and peer-learning. Having instructors who are close in age may allow for more open, honest discussions that promote ethical understanding due to the lack of hierarchy.	[51, 55, 59, 75, 77, 103–110]
Strong role modelling	Good role models who demonstrate ethical behaviour and good professional conduct consistently at work promote the success of ethics training.	[84]
*Curricular Design and Implementation*		
Clear learning objectives	Clear objectives guide learning and assessment.	[50–52, 86]
Allow for preparatory work	Students should be given learning materials early.	[75]
Reflective practice	This refers to good attitudes on the part of the student to engage in reflection, such as through the use of narratives.	[47, 56]
Practice-oriented	The programs should also be practice-oriented and relevant to doctors, such as by highlighting ethical issues faced in real life.	[51, 52, 82, 111]
*Support from Host Institute*		
Training programs for teachers	This includes teacher workshops to assist teachers in developing curricula and acquiring appropriate and relevant teaching skills.	[70]
Devoted educational or health institute, manpower and resources	This may include dedicated ethics experts responsible for teaching, and expert input in the design of curricula.	[64, 70, 79]
**Barriers**	**Elaboration**	**References**
*Learning Environment*		
Poor role models	This may include a culture of bullying and other unethical behaviour exhibited by negative role models.	[61]
*Curricular Design and Implementation*		
Lack of structured curricula	This may lead to important topics not being identified or covered. This could also be due to curricular crowding leading to sacrifices in the ethics curriculum.	[79, 80, 112]
Lack of time and/or opportunity for formal ethics and professionalism instruction	Lack of time was identified as a key limitation for tutors to provide teaching and for students to attend such teaching due to competing demands.	[52, 55, 59, 60, 73, 79, 83, 96–98]
Difficulties in adapting and improving curricula in response to increased sensitivity to ethical concerns	This may lead to outdated curricula.	[103,113]
Lack of an agreed framework that ethics curricula can be designed from and adapted to local settings	This may thus lead to difficulty in adapting curricula to be relevant to the unique ethics situations in different hospitals or different specialties.	[62, 81]
*Barriers from Host Institute*		
Unsupportive institutional culture towards ethics teaching	This may result in having unwilling, underprepared, undertrained teachers	[51, 79, 87, 96–98, 103, 114]
*Learner Factors*		
Poor attitude and resistance to learning	This refers to students who do not seek to improve or are unwilling to be open to ethical discussions or challenge their current understandings and perceptions.	[51, 83, 97, 98]

Believing that new learners often “do not appreciate the practical side of ethical conflicts as they have had limited exposure to clinical medicine or have not yet fully formed a professional identity with its associated values,” Grace and Kirkpatrick [68] piloted ethical vignettes and ethical reasoning technique to acculturate ethical thinking into practice. Howard, McKneally [84]’s study of surgical resident’s attitudes towards ethics teaching revealed a general sense of being poorly prepared and relatively inexperienced for case discussions and practical application of ethical issues.

Carrese, McDonald [60] and Chandra, Ragesh [69] also note that even in the event that ethical issues did arise, they were poorly modelled and rarely used as teaching moments.

#### Assessment tools

Assessment tools comprise the type of assessment method employed, corresponding domains assessed and their pros and cons (Table 6). These assessment methods may be mapped onto the Miller’s pyramid of clinical competency [131].

**Table 6:** Types of assessment methods, domains assessed, advantages, disadvantages

	Assessment methods	Domains assessed	Advantages	Disadvantages
**May be integrated into one of the methods that follow:**	**Clinical scenarios** [72, 115–119]	Identification of ethical issues Creation of a plan to navigate the ethical issue Rationalisation of decision with ethical principles, moral values Real-life anecdotes	Application in ‘real life’ scenarios without direct observation	Subject to varied interpretations
**Knows**	**MCQs** [72, 120–122] [123, 124] [89]	Assessment of learner’s ethical knowledge Comparison of knowledge before and after teaching Clinical scenario-based MCQ	Could be employed as formative and summative assessments Unbiased Trustworthy Less time needed for grading and picks up areas for improvement	Only looks at content knowledge Tough to present clinical situations in a practical, multi-perspective way
**Knows, Knows How**	**Essays** [72, 121, 124–126]	Assessment of knowledge application through a clinical scenario-based essay	Could be employed as formative and summative assessments	Not able to evaluate holistically
**Knows, Knows How**	**SAQs** [73, 126]	Evaluation of knowledge Allowance of deeper reflections and analysis assessments	Focus on distinct areas Able to identify areas for improvement	Inability to apply knowledge effectively Takes a lot of time for both student and teacher
**Knows How, Shows How**	**Debates** [119]	Includes different stakeholder roles	Offers a relevant clinical context	Focuses upon assessing intermediate/ advanced skills and abilities
**Shows How, Does**	**Observations** [72, 120, 125, 127, 128] [126, 129, 130]	May be incorporated as part of an Objective Structured Clinical Examination (OSCE) or evaluation in clinical settings May include a 360-degree evaluation Evaluation of ability to apply content, identification of ethical concerns, ability to analyse and rationalise decisions Individualised feedback from patients and/or simulated patients, tutors and medical professionals	Identifies areas for improvement in clinical/practical settings Identifies biases, lapses in professionalism and deficiencies with techniques Able to provide instant feedback Able to offer productive educational experiences Encourages the learning of knowledge in relevant clinical situations Facilitates longitudinal assessment Reliability amongst inter-raters	Inadequate predictive validity Requires a lot of resources (e.g. time, staff) Subjectivity in simulated patients
**Not specified**	**Self-assessment** [128]	Portfolios provide a longitudinal perspective Evaluation of ability to apply content, identification of ethical concerns, ability to analyse and rationalise decisions	Allows for reflection Popular amongst users and institutions Accurately assesses competencies and learning Good for self-driven learners Learning is documented Various media input Feedback from various stakeholders improves validity	Tough to establish compliance Training is needed

### Stage 5: Discussion and synthesis of SSRs in SEBA

A review of the results and consultation with local educationalists, clinicians and researchers experienced in medical ethics teaching and assessment reiterated the completeness of this review. The narrative produced was guided by the STORIES (Structured approach to the Reporting In healthcare education of Evidence Synthesis) statement [38] and Best Evidence Medical Education (BEME) Collaboration guide [39].This novel review of teaching and assessment of ethics amongst physicians reveals a number of insights. Here we list some of the key findings for ease of reference and will delve into three areas of particular interest.

- The common objective across most ethics programs is to improve awareness of ethical principles and skills in resolving ethical dilemmas tactfully and professionally. More recent articles however focused on changing practice, shaping attitudes and meeting social and professional obligations.
- Recent accounts of teaching and assessing ethics reveal the impact of context and speciality related influences.
- The core elements of most programs concerned the four principles of autonomy, beneficence, non-maleficence and justice; the doctor- patient relationship; communication; and end of life care.
- Speciality or context specific information contents include research ethics; speciality related topics; trainee related considerations and social and or institutional interactions.
- There were a number of approaches employed to teach ethics yet all were focused upon providing learners with an opportunity to apply their knowledge in a variety of ways, ranging from optional participation in group discussions to guided case discussions and reflections.
- Factors facilitating ethics education and assessments were a structured program, a nurturing culture and a safe environment to discuss concerns and enquiries.
- Important in ethics training are role modelling, case-based discussions and instruction on ethical sensitivity and resolving ethical issues.
- There is a general lack of assessment methods.

While there are inherent differences to each of the training programs, they may be seen to lie on a continuum of guiding the learner from knowledge building to practice and ultimately to nurturing the learner’s professional identity. Indeed, many programs seek to prepare learners for their societal responsibilities [49, 60, 70–76, 85] and their membership to their ‘community of practice’ [69, 70]. This would be consistent with Cruess, Cruess [131]’s “Is” level at the apex of their amended Miller’s pyramid. With this in mind, evidence for this posit is visible from the contents and manner that ethics education is taught.

Careful study of the longitudinal nature of training programs, the presence of refresher sessions and/or sessions involving ‘core’ topics such as autonomy, beneficence, non-maleficence, justice, end of life care,the doctor patient relationship and the duty of care suggests a reinforcement of prevailing knowledge [48, 50, 51, 56, 57, 59, 67, 73, 81, 82, 86, 97, 98]. The introduction of more specialised speciality, clinical and research content suggests a layering of new knowledge and experiential learning. This process of building on prevailing knowledge evidences the longitudinal nature of training that would seem to build on training received in medical school and efforts to deepen appreciation of ethical issues in the clinical setting. This is also evidenced by the methods used to train the learners. Here didactic lectures, online videos and bedside ethics discussions give way to case discussions and presentations, allowing the learner to build their knowledge and confidence and apply their knowledge and skills in addressing the ethical issues [58, 69, 74, 78, 86, 99].

These considerations also highlight the vertical aspect of the spiral curriculum employed by most programs and raise the importance of knowledge and skills assessments. Evidence that ethical training is introduced at specific stages of practice such as during postings where end of life care is especially relevant, or where discussions of withdrawing and withholding life sustaining treatment, such as intensive care placements, suggest horizontal integration of the ethics training programs.

The presence of a spiral curriculum that seeks to build on prevailing knowledge and integrate context specific learning highlights two considerations. The first is the use of pertinent assessments to determine progress to the next stage of the training and the second is the support of the program by the host organizsation.

Training should be followed by assessments to ensure that knowledge has been effectively assimilated and applied appropriately, and to facilitate micro-credentialling, as suggested by Norcini [132]. In tandem with this, there is also the need to establish clear Entrustable Professional Activities (EPA) s in ethics education which, at present, will require further research and consideration given the diversity of practice, specialities, socio-cultural considerations and learner variability in terms of their prevailing knowledge, skills, attitudes and experience [133]. The need for a longitudinal assessment process as a part of an education portfolio and their impact on the development of professional identity formation (PIF) also demands closer scrutiny [131, 134].

Here, learning portfolios will allow seamless integration between ethics training in undergraduate and postgraduate training [51, 83, 97, 98] and would be in keeping with the notion of ethics training being part of a longitudinal training experience [4, 135] that nurtures PIF [131, 134]. Portfolios not only serve as a valuable assessment modality for longitudinal evaluation of ethical competency but also promotes continuous self-learning through the recognition of knowledge deficits while reinforcing good behaviour [63, 136–143].

Yet an effective ethics training program requires support fromresidency programs, healthcare institutes and educational institutes through the allocation ofallocating dedicated resources, manpower and faculty training [64, 70, 79]. The host organisation must orchestrate this training and provide careful oversight of the program's trajectory. Perhaps just as important is that there are efforts to ensure that clinicians acknowledge and adopt their roles and responsibilities in their ‘communities of practice’ [144]. The topics chosen should be practical and feasibly covered within the limited time allotted yet be relevant to clinical practice [52, 55, 59, 60, 73, 79, 83, 96–98].

The programs and host organisations must also instil a nurturing ethical climate through the dissemination of core values and introduction of infrastructure that “proactively incorporates these values in the daily life of the healthcare organi[z]ations” [145]. An ethical climate would aid in professional identity formation [131, 134, 146].

## Limitations

Whilst it was our intention to appreciate the range of available literature on ethics education in postgraduate medical education, it is evident that each paper could be studied in greater depth. This limitation is mainly due to incomplete reporting of the current training approaches and their curriculum, as well as the way in which the programs are is carried out and evaluated.

Furthermore, the range of selected articles chosen originates from papers that were largely written in North America and Europe. This limits the applicability of these findings, as the different cultures across the different geographical boundaries are not accounted for.

However, despite these limitations, this scoping review was carried out with the necessary rigour and transparency advocated by Arksey and O’Malley [21], Pham, Rajic [26], and Levac, Colquhoun [147]. The use of Endnote, a bibliographic manager, ensured that all the citations from the different databases were properly accounted for.

## Conclusion

We believe the analysis of our findings in this scoping review will be relevant to educators and program designers in postgraduate medical settings around the world. However, the lack of consensus and difference in perspectives regarding the approach, content and quality assessments as well as the need to explore the inherent link amongst ethics, communication and professionalism [63, 148, 149] justifies inclusion of programs focused on enhancing communication skills and professionalism in medicine. In addition, more needs to be done to research on establishing EPAs in ethics amidst the diverse characteristics of learners, their settings and their levels of experience as well as the particular healthcare system and culture that they practice in. Research should also look into portfolio design, implementation and assessment of PIF and micro-credentialling in ethics practice in the postgraduate setting.

## Supplementary Information

**Additional file 1.** Full PubMed Search Strategy.

**Additional file 2.** Tabulated Summaries for Teaching of Ethics.

**Additional file 3.** Tabulated Summaries for Assessing of Ethics.

## Data Availability

All data generated or analysed during this review are included in this published article [and its additional files].

## Abbreviations

COVID-19: Coronavirus disease 2019
EPA: Entrustable professional activities
SEBA: Systematic evidence-based approach
SSR: Systematic scoping review
YLLSoM: Yong Loo Lin School of Medicine
NUS: National University of Singapore
NCCS: National Cancer Centre Singapore
PICOS: Population, Intervention, Comparison and Outcome
STORIES: Structured approach to the reporting in healthcare education of evidence synthesis
BEME: Best evidence medical education
PIF: Professional identity formation
