# Supplementary material for: Postgraduate ethics training programs: a systematic scoping review
Source: BMC Med Educ. 2021 Jun 9;21:338. doi: 10.1186/s12909-021-02644-5 (PMC8188952; doi:10.1186/s12909-021-02644-5)
Supplement: Supplementary file 1 — Additional file 1. Full PubMed Search Strategy. [file 12909_2021_2644_MOESM1_ESM.pdf]

Additional File 1: <u>Full PubMed Search Strategy</u>

1) PubMed 523

Concept	Subject Headings (MeSH)	Keywords [Title/Abstract]
Ethics	“Code of ethics”[MeSH] OR “Ethics, Medical”[MeSH]	Ethic[tiab] OR ethics[tiab] OR ethical[tiab]
Doctors	Physicians[MeSH] OR “Clinical Clerkship”[MeSH]<br><br>"Education, Medical"[Mesh]	Physician[tiab] OR Physicians[tiab] OR resident[tiab] OR residents[tiab] OR residency[tiab] OR residencies[tiab] OR practice[tiab] OR practitioner[tiab] OR practitioners[tiab] OR doctor[tiab] OR doctors[tiab] OR houseman[tiab] OR housemanship[tiab] OR housemen[tiab] OR “medical officer”[tiab] OR “medical officers”[tiab]
Assessment		Assess[tiab] OR Assessment [tiab] OR Feedback[tiab] OR questionnaire[tiab] OR evaluation[tiab] OR “Multisource feedback” [tiab]
Tools		Tool[tiab] OR tools[tiab] OR Instrument[tiab] OR Rubric[tiab] OR Rubrics[tiab] OR Questionnaire[tiab] OR questionnaires[tiab] OR survey[tiab] OR surveys[tiab]
Medical Education		“Education, medical”[MeSH]

(“Code of ethics”[MeSH] OR “Ethics, Medical”[MeSH] OR Ethic[tiab] OR ethics[tiab] OR ethical[tiab]) AND (Physicians[MeSH] OR “Education, Professional”[MeSH] OR “Clinical Clerkship”[MeSH] OR Physician[tiab] OR Physicians[tiab] OR resident[tiab] OR residents[tiab] OR residency[tiab] OR

residencies[tiab] OR practice[tiab] OR practitioner[tiab] OR practitioners[tiab] OR doctor[tiab] OR doctors[tiab] OR houseman[tiab] OR housemanship[tiab] OR housemen[tiab] OR “medical officer”[tiab] OR “medical officers”[tiab]) AND (Assess[tiab] OR Assessment [tiab] OR Feedback[tiab] OR questionnaire[tiab] OR evaluation[tiab] OR “Multisource feedback” [tiab])

Concept on Medical ethics
"Ethics, Medical/education"[Mesh] OR ethic[tiab] OR ethics[tiab] OR ethical[tiab]

Concept on ‘population’
(“Physicians[MeSH] OR “Clinical Clerkship”[MeSH] OR "Education, Medical/methods"[Mesh] OR Physician[tiab] OR Physicians[tiab] OR resident[tiab] OR residents[tiab] OR residency[tiab] OR residencies[tiab] OR practice[tiab] OR practitioner[tiab] OR practitioners[tiab] OR doctor[tiab] OR doctors[tiab] OR houseman[tiab] OR housemanship[tiab] OR housemen[tiab] OR “medical officer”[tiab] OR “medical officers”[tiab])

Concept on Teaching
"Teaching/methods"[Mesh] OR "Education, Medical/methods"[Mesh] OR "Curriculum"[Mesh] OR “Program Development"[Mesh] OR curricula[tiab] OR curriculum[tiab] OR teaching[tiab] OR teachings[tiab] OR teach[tiab] OR teacher[tiab] OR teachers[tiab] OR tutor[tiab] OR tutors[tiab] OR tutoring[tiab]

Concept on Assessment
("Program Evaluation"[Mesh] OR "Educational Measurement"[Mesh] OR "Surveys and Questionnaires"[Mesh] OR "Feedback"[Mesh] OR Assess*[tiab] OR Feedback[tiab] OR questionnaire[tiab] OR evaluat*[tiab] OR “Multisource feedback” [tiab])

"Hospitals, Teaching"[Mesh]

2) Embase 5849

('education, medical, graduate'/exp OR 'education, medical'/exp OR 'clinical clerkship'/exp OR 'medicine'/exp OR 'medical education':ti,ab)

AND ('physician'/exp OR 'clinical education'/exp OR Physician:ti,ab OR Physicians:ti,ab OR resident:ti,ab OR residents:ti,ab OR residency:ti,ab OR residencies:ti,ab OR practice:ti,ab OR practitioner:ti,ab OR practitioners:ti,ab OR doctor:ti,ab OR doctors:ti,ab OR houseman:ti,ab OR housemanship:ti,ab OR housemen:ti,ab OR “medical officer”:ti,ab OR “medical officers”:ti,ab)

AND ('medical ethics'/exp OR ethic:ti,ab OR ethics:ti,ab OR ethical:ti,ab)

AND ('assessment'/exp OR feedback:ti,ab OR evaluation:ti,ab OR 'questionnaire'/exp OR 'questionnaire':ti,ab OR 'questionnaires':ti,ab OR 'tool'/exp OR tool:ti,ab OR instrument:ti,ab OR rubric:ti,ab)

3) Cochrane 287

(MeSH descriptor: [Code of ethics] explode all trees OR MeSH descriptor: [Ethics, Medical] explode all trees OR (Ethic OR ethics OR ethical):ti,ab)

AND (MeSH descriptor: [Physicians] explode all trees OR MeSH descriptor: [Education, Professional] explode all trees OR MeSH descriptor: [Clinical Clerkship] explode all trees OR (Physician OR Physicians OR resident OR residents OR residency OR residencies OR practice OR practitioner OR practitioners OR doctor OR doctors OR houseman OR housemanship OR housemen OR “medical officer” OR “medical officers”):ti,ab)

AND (MeSH descriptor: [Educational Measurement] OR MeSH descriptor: [Surveys and Questionnaires] OR MeSH descriptor: [Formative Feedback] OR (Assess OR Assessment OR Feedback OR questionnaire OR evaluation OR feedback OR “Multisource feedback”):ti,ab

AND (Tool OR tools OR Instrument OR Rubric OR Rubrics Questionnaire OR questionnaires OR survey OR surveys OR Criteria):ti,ab)

AND (MeSH descriptor: [Education, Medical, Graduate] explode all trees OR MeSH descriptor: [Clinical Clerkship] explode all trees OR MeSH descriptor: [Medicine] explode all trees OR MeSH descriptor: (Medicine OR “medical education”):ti,ab)

Concept	Subject Headings (MeSH)	Keywords [Title/Abstract]
Ethics	MeSH descriptor: [Code of ethics] explode all trees OR MeSH descriptor: [Ethics, Medical] explode all trees	(Ethic OR ethics OR ethical):ti,ab
Doctors	MeSH descriptor: [Physicians] explode all trees	(Physician OR Physicians OR resident OR residents OR residency OR

	OR<br>MeSH descriptor: [Education, Professional] explode all trees<br>OR<br>MeSH descriptor: [Clinical Clerkship] explode all trees	residencies OR practice OR practitioner OR practitioners OR doctor OR doctors OR houseman OR housemanship OR housemen OR “medical officer” OR “medical officers”):ti,ab
Assessment	MeSH descriptor: [Educational Measurement] OR MeSH descriptor: [Surveys and Questionnaires] OR MeSH descriptor: [Formative Feedback]	(Assess OR Assessment OR Feedback OR questionnaire OR evaluation OR feedback OR “Multisource feedback”):ti,ab
Tools		(Tool OR tools OR Instrument OR Rubric OR Rubrics Questionnaire OR questionnaires OR survey OR surveys OR Criteria):ti,ab
Medical Education	MeSH descriptor: [Education, Medical, Graduate] explode all trees<br>OR<br>MeSH descriptor: [Clinical Clerkship] explode all trees<br>OR<br>MeSH descriptor: [Medicine] explode all trees	(Medicine OR “medical education”):ti,ab

4) ERIC 25

(MAINSUBJECT.EXACT("ethics") OR ti(Ethic OR ethics OR ethical) OR ab(Ethic OR ethics OR ethical)) AND (MAINSUBJECT.EXACT("Physicians") OR MAINSUBJECT.EXACT("Family physicians") OR MAINSUBJECT.EXACT("Professional education") OR ti(Physician OR Physicians OR resident OR residents OR residency OR residencies OR practice OR practitioner OR practitioners OR doctor OR doctors OR houseman OR housemanship OR housemen OR “medical officer” OR “medical officers” OR “clinical clerkship”) OR ab(Physician OR Physicians OR resident OR residents OR residency OR residencies OR practice OR practitioner OR practitioners OR doctor OR doctors OR houseman OR housemanship OR housemen OR “medical officer” OR “medical officers” OR “clinical clerkship”)) AND (ti(Assess OR Assessment OR Feedback OR questionnaire OR evaluation OR feedback OR “Multisource feedback”) OR ab(Assess OR Assessment OR Feedback OR questionnaire OR evaluation OR feedback OR “Multisource feedback”)) AND (ti(Tool OR tools OR Instrument OR Rubric OR Rubrics OR Questionnaire OR questionnaires OR survey OR surveys OR Criteria) OR ab(Tool OR tools OR Instrument OR Rubric OR Rubrics OR Questionnaire OR questionnaires OR survey OR surveys OR Criteria)) AND MAINSUBJECT.EXACT("Medicine") OR ti(Medicine OR “medical education” OR “graduate medical education” OR “clinical clerkship”) OR ab(Medicine OR “medical education” OR “graduate medical education” OR “clinical clerkship”))

Concept	Subject Headings (MeSH)	Keywords [Title/Abstract]
Ethics	MAINSUBJECT.EXACT("ethics")	Ethic OR ethics OR ethical
Doctors	MAINSUBJECT.EXACT("Physicians") OR MAINSUBJECT.EXACT("Family physicians") OR MAINSUBJECT.EXACT("Professional education")	Physician OR Physicians OR resident OR residents OR residency OR residencies OR practice OR practitioner OR practitioners OR doctor OR doctors OR houseman OR housemanship OR housemen OR “medical officer” OR “medical officers” OR “clinical clerkship”
Assessment		Assess OR Assessment OR Feedback OR questionnaire OR evaluation OR feedback OR “Multisource feedback”
Tools		Tool OR tools OR Instrument OR Rubric OR Rubrics Questionnaire OR questionnaires OR survey OR surveys OR Criteria

Medical Education	MAINSUBJECT.EXACT("Medicine")	Medicine OR “medical education” OR “graduate medical education” OR “clinical clerkship”

5) Scopus 7315

(SUBJAREA (medi) AND (ethics W/0 medical) OR (ethics W/0 clinical)) OR TITLE-ABS (“ethic” OR “ethics” OR “ethical”) AND (SUBJAREA (medi) AND (“Physicians” OR (education W/0 professional) OR “clinical clerkship”)) OR TITLE-ABS (“Physician” OR “Physicians” OR “resident” OR “residents” OR “residency” OR “residencies” OR “practice” OR “practitioner” OR “practitioners” OR “doctor” OR “doctors” OR “houseman” OR “housemanship” OR “housemen” OR “medical officer” OR “medical officers”) AND TITLE-ABS (Assess OR Assessment OR Feedback OR questionnaire OR evaluation OR feedback OR “Multisource feedback”) AND TITLE-ABS (Tool OR tools OR Instrument OR Rubric OR Rubrics OR Questionnaire OR questionnaires OR survey OR surveys OR Criteria) AND (SUBJAREA (medi) AND ("Graduate medical education" OR "medical education" OR (medicine W/0 education) OR "clinical clerkship" OR "medicine") OR TITLE-ABS (“Medicine” OR “medical education”)

Concept	Subject Headings (MeSH)	Keywords [Title/Abstract]
Ethics	(SUBJAREA (medi) AND (ethics W/0 medical) OR (ethics W/0 clinical)))	TITLE-ABS (“ethic” OR “ethics” OR “ethical”)
Doctors	(SUBJAREA (medi) AND (“Physicians” OR “clinical clerkship”))	TITLE-ABS (“Physician” OR “Physicians” OR “resident” OR “residents” OR “residency” OR “residencies” OR “practice” OR “practitioner” OR “practitioners” OR “doctor” OR “doctors” OR “houseman” OR “housemanship” OR “housemen” OR “medical officer” OR “medical officers”)

Assessment		TITLE-ABS (Assess OR Assessment OR Feedback OR questionnaire OR evaluation OR feedback OR "Multisource feedback")
Tools		TITLE-ABS (Tool OR tools OR Instrument OR Rubric OR Rubrics OR Questionnaire OR questionnaires OR survey OR surveys OR Criteria)
Medical Education	(SUBJAREA (medi) AND ("Graduate medical education" OR "medical education") OR (medicine W/0 education) OR "clinical clerkship" OR "medicine")	TITLE-ABS ("Medicine" OR "medical education")

6) PSYCHINFO 154

Concept	Subject Headings (MeSH)	Keywords [Title/Abstract]
Ethics	exp bioethics/ OR exp ethics/	(ethic OR ethics OR ethical).ti,ab.
Doctors	exp physicians/	(Physician OR Physicians OR resident OR residents OR residency OR residencies OR practice OR practitioner OR practitioners OR doctor OR doctors OR houseman OR

		housemanship OR housemen OR medical officer OR medical officers OR professional education OR clinical clerkship).ti,ab.
Assessment		(Assess OR Assessment OR Feedback OR questionnaire OR evaluation OR feedback OR “Multisource feedback”).ti,ab.
Tools		(Tool OR tools OR Instrument OR Rubric OR Rubrics Questionnaire OR questionnaires OR survey OR surveys OR Criteria).ti,ab.
Medical Education	exp medical education/	(Medicine OR medical education* OR clinical clerkship).ti,ab.

exp bioethics/ OR exp ethics/ OR (ethic OR ethics OR ethical).ti,ab AND (exp physicians/ OR (Physician OR Physicians OR resident OR residents OR residency OR residencies OR practice OR practitioner OR practitioners OR doctor OR doctors OR houseman OR housemanship OR housemen OR medical officer OR medical officers OR professional education OR clinical clerkship).ti,ab) AND ((Assess OR Assessment OR Feedback OR questionnaire OR evaluation OR feedback OR Multisource feedback).ti,ab) AND ((Tool OR tools OR Instrument OR Rubric OR Rubrics Questionnaire OR questionnaires OR survey OR surveys OR Criteria).ti,ab.) AND (exp medical education/ OR (Medicine OR medical education* OR clinical clerkship).ti,ab)
