# Supplementary material for: Postgraduate ethics training programs: a systematic scoping review
Source: BMC Med Educ. 2021 Jun 9;21:338. doi: 10.1186/s12909-021-02644-5 (PMC8188952; doi:10.1186/s12909-021-02644-5)
Supplement: Supplementary file 2 — Additional file 2. Tabulated Summaries for Teaching of Ethics. [file 12909_2021_2644_MOESM2_ESM.pdf]

Additional File 2: **Tabulated Summaries for Teaching of Ethics**

Title	Author/Year	Type of study	MERSQI	COREQ	Methodology	Purpose of study	Key findings	Proposed solutions
A window of opportunity: Ethics and professionalism in the obstetrics and gynecology clerkship	Belling & Coulehan 2006	Case study	NA	NA	At Stony Brook School of Medicine, we utilize these concepts by incorporating a structured ethics and professionalism exercise into the 3rd-year obstetrics-gynecology clerkship. Students utilize a team approach in a Medicine in Contemporary Society exercise that requires them to work up and present a patient case from an ethical, social, cultural, legal, and/or economic perspective in an inter-active setting.	In this article, we describe our experience over a 9-year period employing this exercise with nearly 1,300 students.	In 12 years, 1,272 students (282 groups) completed the clerkship. Although presentations sometimes address two or more issues, Table 1 indicates the major or primary issue listed for each presentation. As can be seen, informed consent, termination of pregnancy, maternal-fetal conflict, right to refuse treatment, and assisted reproduction were the most frequently addressed individual topics. These echo the experience of Cain et al., aside from more frequent discussions of assisted reproduction, which reflects the increased usage of these technologies since the early 1990s. Most discussions involved obstetrics patients (80%), and about half of these (40% overall) concerned issues specific to obstetrics such as maternal-fetal conflicts and abortion; whereas in the other half, the contextual issues were nonspecific but more complex owing to the pregnancy. A relatively small percentage of student-selected cases (15%) focused primarily on social, cultural, or economic issues such as barriers in communicating with non-English-speaking patients or cultural differences in gender relationships (e.g., a pregnant woman who abdicates all decision making to her husband). However, even when an ethical issue was considered primary, students identified socioeconomic and cultural factors as coissues in the majority of cases: for example, insurance coverage (an economic concern) for in vitro fertilization or abortion (ethical concerns).	In summary, the MCS window of opportunity provides our students with a “booster shot” of personal, ethical, and social awareness in obstetrics-gynecological care during the clerkship experience, a period during which they are primed for rapid professional growth; and it helps them resist the reductionistic approach to obstetrics and gynecology promoted by today’s fast-paced hospital practice.

What is empathy, and how can it be promoted during clinical clerkships?	Benbassat & Baumal 2011	Descriptive	NA	NA	The authors describe patient interviewing style that facilitates empathy and some practice habits that interfere with it.	The ability of medical students to empathize often declines as they progress through the curriculum. This suggests that there is a need to promote empathy toward patients during the clinical clerkships. In this article, the authors attempt to identify the patient interviewing style that facilitates empathy and some practice habits that interfere with it.	The authors maintain that (1) empathy is a multistep process whereby the doctor's awareness of the patient's concerns produces a sequence of emotional engagement, compassion, and an urge to help the patient; and (2) the first step in this process—the detection of the patient's concerns—is a teachable skill. The authors suggest that this step is facilitated by (1) conducting a "patient-centered" interview, thereby creating an atmosphere that encourages patients to share their concerns, (2) enquiring further into these concerns, and (3) recording them in the section traditionally reserved for the patient's "chief complaint." Some practice habits may discourage patients from sharing their concerns, such as (1) writing up the history during patient interviewing, (2) focusing too early on the chief complaint, and (3) performing a complete system review.	The authors conclude that sustaining empathy and promoting medical professionalism among medical students may necessitate a change in the prevailing interviewing style in all clinical teaching settings, and a relocation of a larger proportion of clinical clerkships from the hospital setting to primary care clinics and chronic care, home care, and hospice facilities, where students can establish a continuing relationship with patients.
The Teaching of Ethics and Professionalism in Plastic Surgery Residency: A Cross-Sectional Survey	Bennett et al. 2017	Quantitative	15.5	NA	A 21-question survey was sent from the American Council of Academic Plastic Surgeons meeting to 180 plastic surgery program directors and coordinators via email. Survey questions inquired about practice environment, number of residents, presence of a formal ethics training program, among others. Binary regression was used to determine if any relationships existed between categorical variables, and Poisson linear regression was used to assess relationships between continuous variables. Statistical significance was set at a P value of 0.05.	The ethical practice of medicine has always been of utmost importance, and plastic surgery is no exception. The literature is devoid of information on the teaching of ethics and professionalism in plastic surgery. In light of this, a survey was sent to ascertain the status of ethics training in plastic surgery residencies.	A total of 104 members responded to the survey (58% response rate). Sixty-three percent were program directors, and most (89%) practiced in academic settings. Sixty-two percent in academics reported having a formal training program, and 60% in private practice reported having one. Only 40% of programs with fewer than 10 residents had ethics training, whereas 78% of programs with more than 20 residents did. The odds of having a training program were slightly higher (odds ratio, 1.1) with more residents ($P = 0.17$).	Despite the lack of information in the literature, formal ethics and professionalism training does exist in many plastic surgery residencies, although barriers to implementation do exist. Plastic surgery leadership should be involved in the development of standardized curricula to help overcome these barriers.
Teaching ethics of psychopharmacology research in psychiatric	Beresin et al. 2003	Review	NA	NA	We reviewed recent literature on ethical issues in clinical research and on medical education in bioethics	We consider major contemporary ethical issues in clinical psychiatric research, with an emphasis on psychopharmacology, and implications of addressing them	This report considers: (1) an overview of current training; (2) perceived needs and rationales for training in research ethics, (3) recommended educational	We recommend that psychiatric training include education about ethical aspects of clinical research, with a particular emphasis on

residency training programs						within residency training programs.	content and methods; (4) issues that require further study (including demonstration of acquired knowledge, practice issues, and the treatment versus-investigation misconception); and (5) conclusions. Recommended components of residency training programs include basic ethical principles; scientific merit and research design; assessment of risks and benefits; selection and informed consent of patient-subjects; and integrity of the clinical investigator, including definition of roles, conflicts-of-interest, and accountability. Evaluation of educational effectiveness for both trainees and faculty is a recommended component of such programs.	psychopharmacology. These activities can efficiently be incorporated into teaching of other aspects of bioethics, research methods, and psychopharmacology. Such education early in professional development should help to clarify roles of clinicians and investigators, improve the planning, conduct and reporting of research, and facilitate career development of much-needed clinical investigators in psychiatry.
Core strategies for the development of a clinical neuroethics education program for medical residents in the clinical neurosciences.	Buchman et al. 2009	Qualitative	NA	21	In this paper we describe a new education program in ethics for medical residents in the clinical neurosciences – Clinical Neuroethics – that integrates teaching material from medicine, neuroscience, and biomedical ethics. In this context, (1) we explore the importance of ethics education and training in the clinical neurosciences; and (2) we describe our approach to complementing existing Ethics requirements established for example, in Canada, by professional organizations such as the Royal College of Physicians and Surgeons of Canada (RCPSC) and the British Columbia College of Physicians and Surgeons (BCCPS). The program is still in its nascent phases and evolves with continual input and evaluation by participating residents from Neurology, Neurosurgery and Psychiatry.	The emergent field of neuroethics in which we ground our efforts lies at the intersection of novel developments in neurotechnology and the implications of those developments for society, in the laboratory, the home, the courthouse, the classroom, and the clinic. Neuroethics integrates research ethics in the biomedical sciences and public health ethics with brain science and neuro- and moral philosophy. Scholars in neuroethics study the relevant ethical, legal, social, cultural, policy, and clinical challenges associated with new ways to think about, maintain and manipulate brain function.	We describe core components of the program here, including journal clubs on tough neuro-clinical cases, seminars on cutting edge topics in neuroethics, and opportunities for residents to innovate in research. We compare and contrast the relative strengths and limitations of the strategies implemented, and present a vision for next steps based on what we have learned to date.	In reflecting upon the guiding principles for the Clinical Neuroethics program and pilot experiences, we summarize our approach to shaping education in neuroethics as follows: We start with the learner's/resident's point of view and use experiential bridges between the learner's experience, ways of knowing, and educational goals. We continually incorporate resident direction and feedback. We aim to develop a responsive curriculum that is relevant and meaningful to the residents' own practice and ethical challenges. Creativity and flexibility are core factors in ensuring a close alignment of ethics instruction, neuroscience advancements, and clinical practice. In an era of constant progress in clinical neuroscience, our ultimate goal mirrors that of medical educators and trainees – parallel progress in clinical neuroethics and quality of care.
Evaluation of	Byrne et al.	Quantitative	14	NA	A cross-sectional, web-based	The objective of the study was to	Of 242 eligible obstetrics-	This study found that a lack of

ethics education in obstetrics and gynecology residency programs	2015				survey was designed in conjunction with a professional survey laboratory at the University of Chicago. The survey was piloted with a convenience sample of clinical medical ethics fellows to assess question content and clarity. The survey was deployed by e-mail to all obstetrics-gynecology residency program directors. Descriptive statistics were used to analyze participant responses. The University of Chicago's Institutional Review Board deemed this study exempt from institutional review board formal review.	assess the current status of ethics education in obstetrics-gynecology residency programs.	gynecology residency program directors, 118 (49%) completed the survey. Most respondents were from university-based programs (n 1⁄4 78, 66%) that were not religiously affiliated (n 1⁄4 98, 83%) and trained 4-6 residents per postgraduate year (n 1⁄4 64, 70%). Although 50% of program directors (n 1⁄4 60) reported having ethics as part of their core curriculum, most programs teach ethics in an unstructured manner. Fifty-seven percent of respondents (n 1⁄4 66) stated their program dedicated 5 or fewer hours per year to ethics. The majority of program directors (n 1⁄4 80, 73%) responded they would like more to a lot more ethics education and believed that ethics education should be required (n 1⁄4 93, 85%) for residents to complete their training. Respondents identified that crowding in the curriculum was a significant barrier to increased ethics training (n 1⁄4 50, 45%) and two-thirds (n 1⁄4 74, 67%) reported a lack of faculty expertise as a moderate barrier to providing ethics education in the residency curriculum.	structured curricula, inadequate faculty expertise, and limited time were important barriers for ethics education in obstetrics-gynecology programs across the nation. Despite these existing challenges, program directors have a strong interest in increasing ethics education in residency training. Therefore, additional resources are needed to assist program directors in enhancing resident ethics education.
Ethics in the teaching of mental health professionals	Callan & Bucky 2005	Descriptive	NA	NA	This article discusses the teaching of ethics to mental health professionals using CSPP-San Diego (Alliant International University) as a model. The authors stress the importance of thoroughly teaching mental health professionals-in-training how to integrate clinical, legal, and eth- ical issues in an organized, systematic fashion.	This article focuses on these two emphases, with initial attention to the teaching of ethics as a part of education and training in psychology at all levels, followed by attention to ethical considerations more generally relevant to all aca- demic settings and relationships therein. In support of both of these concerns, but particularly the former (the teaching of ethics as a part of education and training in psychology), an example of one curricular model, as developed for educating future professional psychologists during their doctoral training, is described.	The California School of Professional Psychology (Alliant International University) San Diego campus has developed a model that requires (a) an ethics course integrated with the student's on-campus supervision in the Fall and Spring semesters at the practicum level, focusing on the American Psychological Association's Ethics Code (2002) and California's Licensing Laws; and (b) an advanced ethics course (in the fourth year of training) that focuses on the integration of ethical and legal issues in clinical practice and providing a knowledge base of sound ethical judgment. Syllabi for these two courses are	It is the position of the authors that mental health professionals-in-training must be well versed in the ethical/legal matters of clinical practice. Indeed, most graduate programs in the field of mental health require formal training in Ethics.

							included.	
Everyday ethics in internal medicine resident clinic: An opportunity to teach.	Carrese et al. 2011	Qualitative	NA	17	This study involved a multi-method qualitative research design combining observation of preceptor-resident discussions with preceptor interviews. The study was conducted in two different internal medicine training programme clinics over a 2-week period in June 2007. Fifty-three residents and 19 preceptors were observed, and 10 preceptors were interviewed. Transcripts of observer field notes and faculty interviews were carefully analysed. The analysis identified several themes of everyday ethics issues and determined whether preceptors identified and taught about these issues.	Being a good doctor requires competency in ethics. Accordingly, ethics education during residency training is important. We studied the everyday ethics-related issues (i.e. ordinary ethics issues commonly faced) that internal medical residents encounter in their out- patient clinic and determined whether teaching about these issues occurred during faculty preceptor–resident interactions.	Everyday ethics content was considered present in 109 (81%) of the 135 observed case presentations. Three major thematic domains and associated sub-themes related to everyday ethics issues were identified, concerning: (i) the Doctor–Patient Interaction (relationships; communication; shared decision making); (ii) the Resident as Learner (developmental issues; challenges and conflicts associated with training; relationships with colleagues and mentors; interactions with the preceptor), and; (iii) the Doctor–System Interaction (financial issues; doctor– system issues; external influences; doctor frustration related to system issues). Everyday ethics issues were explicitly identified by preceptors (without teaching) in 18 of 109 cases (17%); explicit identification and teaching occurred in only 13 cases (12%).	In this study a variety of everyday ethics issues were frequently encountered as residents cared for patients. Yet, faculty preceptors infrequently explicitly identified or taught these issues during their interactions with residents. Ethics education is important and residents may regard teaching about the ethics-related issues they actually encounter to be highly relevant. A better understanding of the barriers to teaching is needed in order to promote education about everyday ethics in the out-patient setting.
Ten-minute snapshots - a team approach to teaching postgraduates about professional dilemmas	Chandra et al. 2017	Qualitative	NA	14	Important scenarios were selected from among these. They included common professional challenges that trainees might face in their day-to-day practice, such as those involving confidentiality, being presented with gifts, boundary violations by patients or their relatives in the form of personal questions, patients' attempts to develop a personal relationship with the health professional, managing interruptions in the clinic and violations of privacy. Some of the other scenarios pertained to explaining medical errors and handling bullying by a senior when it happens in front of patients.	The barriers to learning how to handle professional dilemmas are many. They include the fact that such situations occur infrequently, they catch one unawares, one's peers and teachers do not talk about them and one often tends to brush them under the carpet. The main barrier, of course, is that there is seldom any formal instruction in competencies related to professionalism	Instruction in professionalism is an important part of training in medicine. Professionalism is mostly learnt from role models or with experience, but it is necessary to evolve and assess formal teaching methods that are context – and culture-specific. The importance of cultural factors in training in professionalism has been emphasised	Any evaluation will have to rely only on subjective reports or simulated situations. This training methodology can be evaluated in two ways. One is by assessing the extent to which the perceptiveness of the trainees and the discussions among them become more mature over several sessions. The second is by asking the trainees for written feedback on what they have learnt and how they would handle a similar situation. Finally, the best but most difficult method of evaluation is to see if the trainees handle their day-to-day dilemmas more professionally and ethically in

								the future. This is possible only through direct observation and by obtaining feedback from patients.
Young Physicians' Recall about Pediatric Training in Ethics and Professionalism and Its Practical Utility	Cook & Ross 2013	Quantitative	9	NA	We surveyed a random sample of members of the American Academy of Pediatrics Section on Young Physicians between February and June 2012.	To assess the adequacy of ethics and professionalism education in residency by examining the recollections of young pediatricians in practice.	The majority of young pediatricians reported that ethics and professionalism were taught ad hoc in their training programs. Compared with physicians in practice for >5 years, those in practice for #5 years were significantly more likely to report having had an organized curriculum (72 of 181 [40%] vs 27 of 113 [24%]; P < .01) and that the ethics and professionalism training in their program was adequate (124 of 180 [69%] vs 62 of 113 [55%]). Of the topics encountered in practice by at least two-thirds of pediatricians, more than two-thirds of the respondents stated that residency training adequately prepared them to address issues of consent, privacy, truth-telling, and child abuse/neglect, but less than one-third felt adequately prepared to address conduct on social media and requests for prescriptions by family, friends, and colleagues outside of clinical encounters.	As pediatric residency programs adopt more structured curricula for ethics and professionalism education, issues commonly faced by practitioners should be incorporated.
Teaching and assessing ethics in the newborn ICU	Cummings 2016	Review	NA	NA	Ethics and professionalism education has become increasingly recognized as important and incorporated into graduate medical education. However, such education has remained largely unstructured and understudied in neonatology. Neonatal–perinatal fellowship training programs have generally grappled with how best to teach and assess ethics and professionalism knowledge, skills, and behavior in clinical practice, particularly in light of accreditation requirements, milestones, and competencies.	This article reviews currently available teaching methods, pedagogy, and resources in medical ethics, professionalism, and communication, as well as assessment strategies and tools, to help medical educators and practicing clinicians ensure trainees achieve and maintain competency in neonatology. Finally, the need for consensus and future research in these domains is also highlighted.	Appropriate assessment is crucial to drive further learning. Just as evidence for the best pedagogical methods to teach ethics and professionalism are lacking in neonatology, evidence for optimal strategies for assessment in these areas are also lacking. As discussed above, many neonatal–perinatal training programs and their associated Clinical Competency Committees (CCC) have largely struggled with how best to assess competencies, milestones, and EPAs, particularly those related to ethics and professionalism. Yet, despite existing challenges, assessment in these domains is	A “flipped classroom” approach to teaching medical ethics and professionalism in neonatology may be effective, in which students learn course material online via multimedia modules on their own time, then come to class sessions prepared to engage in a variety of educational activities, including in-depth group discussion, enacted role-play, and simulation. This model would engage students with various preferred learning styles, and enable mastery of both knowledge and practical skills in ethics and professionalism during training when time is scarce. Our

							impossible without some type of measurement.	group is in the process of piloting such an innovative ethics and professionalism curriculum for neonatal–perinatal fellows, as well as developing and validating 2 assessment tools. Outcomes-based research is still needed to determine optimal teaching methods as well as assessment strategies in ethics and professionalism in neonatology to ensure trainees not only achieve competency in these domains, but also to ensure the formation of professional, virtuous clinicians with excellent moral and ethical reasoning skills for the ultimate benefit of the patients and their families.
How we teach ethics and communication during a Canadian neonatal perinatal medicine residency: An interactive experience.	Daboval et al. 2013	Qualitative	NA	16	This article describes the process by which a well-delineated, interactive program to teach ethical reasoning and skillful communication with parents was implemented at the University of Ottawa, Canada.	A revised ethics program implemented in 2009 identified competencies that should be demonstrated at the end of the Neonatal-Perinatal Medicine (NPM) residency. Several seminars were refined while new workshops, problem-based learning in ethics, and a personal portfolio were added.	All teaching strategies were well received based on the average level of satisfaction (5.8 out of 7, SD 0.4). We are now moving forward by formally assessing our program including the impact on knowledge acquisition and behavior.	A dedicated, interactive competency-based neonatal ethics teaching program is vital to support NPM trainees in learning how to integrate ethical thinking with competencies in communication.
Narrative ethics as collaboration: A four-session curriculum	Goodrich et al. 2005	Mixed	6.5	14	To teach this approach, the authors developed a residency curriculum composed of 4 half-day sessions. The curriculum includes narrative theory, narrative analysis, short stories, and residents' writings.	Our goal has been to create a curriculum that residents would find both engaging and clinically relevant. The objectives are that the participants will 1. understand the narrative basis of medicine and the perspectival nature of narrative; 2. appreciate the ethical implications of all aspects of the medical encounter; 3. be able to identify the omnipresence of family systems, social location, and cultural context in formulating clinical action; and 4. use narrative analysis as a means for elucidating how social location and cultural context structure ethical di- lemmas and inform clinical decision making.	Ratings of its helpfulness and interest have been uniformly positive. Residents described the experience as both professionally educational and personally enriching.	This demonstration allows for an intentional opening of space for the perspectives of patients, family members, and colleagues from other disciplines. To teach this approach, the authors developed a residency curriculum composed of 4 half-day sessions. The curriculum includes narrative theory, narrative analysis, short stories, and residents' writings. Ratings of its helpfulness and interest have been uniformly positive. Residents described the experience as both professionally educational and personally enriching.
Ethics and	Goold & Stern	Qualitative	NA	14	Study design is a thematic	The objective of this study is to	Resident and nonresident	Conclusions are that

professionalism: What does a resident need to learn?	2006				analysis of documents, semi-structured interviews, and focus groups conducted in a setting of an academic medical center, Veterans Administration, and community hospital training more than 1000 residents. Participants were 84 informants in 13 specialties including residents, program directors, faculty, practicing physicians, and ethics committees. Thematic analysis identified commonalities across informants and specialties.	describe empirically derived ethics objectives for ethics and professionalism training for multiple specialties.	informants identified consent, interprofessional relationships, family interactions, communication skills, and end-of-life care as essential components of training. Nonresidents also emphasized formal ethics instruction, resource allocation, and self-monitoring, whereas residents emphasized the learning environment and resident-attending interactions.	empirically derived learning needs for ethics and professionalism included many topics, such as informed consent and resource allocation, relevant for most specialties, providing opportunities for shared curricula and resources.
Teaching ethics that honor the patient's and the provider's voice: The role of clinical integrity	Grace, & Kirkpatrick 2018	Qualitative	NA	20	This module on teaching ethics to medical learners involves three steps: presenting background bioethical principle information, teaching the tool to organize discussion of cases, and applying it to six sample clinical scenarios for discussion.	We provide several hypothetical ethical vignettes for practice and discussion using the clinical integrity tool. The article also describes how this module has been implemented in one medical education setting and provides suggestions for educators.	The educational module, including definition of principles and working through a sample case with the five-box technique, has been utilized in multiple graduate medical education settings at one of the author's hospitals [HAK]. These have included teaching a case-based ethics series during morning report and on cases that residents have been included in during the course of Clinical Ethics Committee consults. In addition to this tool, instructions for group discussion and several hypothetical ethical vignettes (Figure 2) were recently presented at a national conference18 where feedback was obtained.	A longitudinal curriculum could ideally address all six topics in one year and would likely take less time per case as the ethical principle review would be shorter as the series progressed. Another format could include a workshop or "boot camp" in which multiple groups are assigned to different scenarios, then present a summary of their discussion and conclusions to the larger group. This allows for direct usage of the tool as well as exposure to a wider variety of the cases. A third format could include a flipped classroom design in which a group of residents presents the case and leads the discussion, with the entire group having conducted the exercise individually in advance.
Ethics education in surgical residency: Past, present, and future	Grossman et al. 2010	Quantitative	12	NA	To quantify and compare teaching methods, articles describing specific ethics educational curricula in residency training programs were categorized into the following 3 distinct teaching modalities: (1) lecture-based curricula; (2) case-based didactics; and (3) integrated lecture/case-based discussion. The survey. A 4-page, multiple-choice, web-based questionnaire modeled from a previously	To improve ethics education in surgery residencies, this article reviews published reports that pertain to specific initiatives in teaching ethics. Moreover, to quantify and analyze the current status of ethics education in surgery residencies, we report data from a nationwide survey of general surgery program directors (PDs). These survey data comprise the first and only known survey of PDs focused solely on ethics education in more	Most of these articles describe the experience of a single institution with 1 teaching methodology; unfortunately, few comparative studies assert that any method is superior to others. Nonetheless, a few such comparative trials have been performed.	In conclusion, the data synthesized in this article demonstrates that surgery residents, both historically and currently, desire ethics education; this ethics education can yield positive results that are both quantifiable and clinically relevant. Although strong data regarding the existing teaching methodologies are not available, we believe that the most stimulating, engaging,

					published Survey, 1 with an introductory e-mail describing the goals of the research, was distributed electronically to PDs of all U.S. general surgery residency programs, as recognized by the American Council for Graduate Medical Education (ACMGE) in 2008.	than a decade.1<br>Finally, by synthesizing the cumulative data, we outline specific proposals to define and enhance ethics education in surgery residency programs.		and relevant educational experience would be achieved via the adoption of a uniform, interactive, and integrated case-based curriculum for all surgery residents.
The Hidden Ethics Curriculum in Two Canadian Psychiatry Residency Programs: A Qualitative Study	Gupta et al. 2016	Qualitative	NA	25	We decided to use qualitative case study as our overall research design because it is appropriate when (1) the phenomenon of interest is ongoing rather than historical and cannot be separated from the context in which it occurs and (2) researchers want to answer "how" and "why" questions in addition to determining what is happening in the situation under study. Specifically, we conducted an instrumental case study because we were using the cases (the programs) to gain insight into the issue of the potential divergences between the formal, informal, and hidden ethics curricula rather than evaluating the specific programs themselves. We also decided to conduct a multiple rather than single-case study because we wanted to gain in-depth understanding of the issue by comparing data from two very different contexts that were likely to shape study participants' experiences and views on ethics training in their Programs.	We aimed to compare and contrast the content of the formal, informal, and hidden ethics curricula in two demographically different postgraduate psychiatry programs (hereafter program 1 and program 2) and to explain potential divergences between the three curricula. To achieve these aims, we compared findings from three sources of data: individual interviews with residents and with faculty and relevant documents from each program. This in-depth qualitative study serves as a useful empirical complement to the existing conceptual work on the hidden curriculum in ethics, which lays<br>out, on theoretical grounds, the high-level values embedded within the culture of the medical profession that motivate the hidden curriculum	Divergences occurred between the curricula for each topic. The nature of these divergences differed according to local program characteristics. Yet, in both programs, choices<br>for action in ethically challenging situations were mediated by<br>a minimum standard of ethics that led individuals to avoid trouble even if this meant their behavior fell short of the accepted ideal.	Effective ethics education in postgraduate psychiatry training will require addressing the hidden curriculum. In addition to profession-wide efforts to articulate high-level values, program-specific action on locally relevant issues constitutes a necessary mechanism for handling the impact of the hidden curriculum.
The hidden curriculum, ethics teaching, and the structure of medical education.	Hafferty & Franks 1994	Qualitative	NA	19	The authors raise questions regarding the widespread calls to intensify the teaching of medical ethics within the medical school curriculum.	In this article we explore the relationship between the formal and the informal teaching of medical ethics during medical training. We also examine-albeit more indirectly-how three beliefs, described below, serve to marginalize ethics in the culture of medicine.	If the arguments advanced above have merit, then it follows that the teaching of ethical principles in the medical school curriculum should be approached and framed quite differently from the teaching of other basic science subjects.	Those who each students need to become more aware of the perceptions of initiates, particularly those at the earliest stages of their training.
Ethics Education in Surgical	Helft et al.. 2009	Literature Review	NA	NA	However, our goal was to summarize the published	We seek to review the literature concerning ethics education in	The few studies in the literature suggest that ethics education,	These findings suggest that ethics education for surgical

Residency Programs: A Review of the Literature					literature specifically pertaining to and focusing on ethics education and training in postgraduate surgical training programs. We conducted a literature review using Medline and Ovid databases. Our initial search retrieved 58 articles using conjunctive searches of the terms “surgery,” “ethics education,” “residents,” and “surgical residents.” We substituted our search using the search terms “clerkship,” “residency,” “medical ethics,” “general surgery residency programs,” “postgraduate surgical residency,” and “graduate medical ethics education.” We excluded articles that discussed professionalism primarily (except those that seemed to pertain specifically to the ethical aspects of professionalism); technical training; issues specific to practicing surgeons, rather than surgical residents; and those otherwise beyond the scope of our selected topic of ethics education within US surgical postgraduate residency programs. A second conjunctive search was performed using “surgery,” “residency,” “end of life care,” “palliative care,” and “communication.” This search retrieved 22 articles. We also searched the references of all identified papers and obtained all published articles, web content, and abstracts that seemed relevant to our topic. Here, we summarize the results of this review.	postgraduate surgical training programs.	when integrated in surgical residency curricula, can lead to measurable improvements in resident-centered outcomes, which include knowledge and confidence in handling ethical dilemmas. These curricula may lead to improvements in patient care.	residents is valuable but that questions regarding the optimal “dose” of ethics education and training as well as the optimal teaching methods would benefit from extended systematic evaluation and inquiry.
Web modules on professionalism and ethics	Hendee et al. 2012	Descriptive	NA	NA	NA	Health care disciplines have always held resolutely to a commitment to professionalism and high ethical standards. With the present emphasis on public accountability, professionalism and ethics are receiving enhanced attention in health care education and practice. A challenge for	NA	The curriculum was developed by the ABR Foundation and included in a request for proposals that was widely distributed. Teams of authors for each of 10 modules were selected from respondents to the request for proposals. As the modules were developed,

						radiologists, radiation oncologists, and medical physicists is to define the scope and depth of knowledge about professionalism and ethics that are necessary for the practice of the disciplines. A further challenge is to develop accessible educational materials that encompass this required knowledge. About 2 years ago, the ABR Foundation decided to address these challenges through the development of an ethics and professionalism curriculum and production of a series of Web-based educational modules that follow the curriculum. Six organizations agreed initially to contribute financially to construction of the curriculum and modules and were later joined by a seventh.		they were reviewed in 3 successive stages, including peer review by members of the ACR Committee on Professionalism and the RSNA-ACR Task Force on an Ethics Curriculum. After revisions were prepared in response to the reviews, the modules were translated into a format compatible with the e-learning platform on which they are mounted. The modules are now available to all who wish to study them.
Integrating bioethics into postgraduate medical education: The university of Toronto model	Howard et al. 2010	Descriptive	NA	NA	To better understand how the University of Toronto model for PGBE compares with programs across Canada, we contacted 11 Canadian medical education centers in February and March 2008 regarding ethics teaching in their residency programs.	We describe our program, initiatives, and research here to share with other educators our model and the lessons we have learned.	Adoption of the CanMEDS framework in 1996 organized PGME in Canada around seven roles: Medical Expert, Communicator, Collaborator, Health Advocate, Manager, Scholar, and Professional. As part of the 2005 revision, ethics competency has been integrated in several areas of the framework: ethics of practice (Medical Expert), ethics of teaching and research (Scholar), ethics in the doctor–patient relationship (Communicator), and team ethics (Collaborator), including confidentiality, resource allocation, and professionalism.	They postulate that centralized support is a key component to ensure the success ofs pecialty-specific bioethics teaching, to reinforce the importance of ethics in medical training, and to ensure it is not overshadowed by other educational concerns.
The formal and informal surgical ethics curriculum: Views of resident and staff surgeons in Toronto	Howard et al. 2012	Qualitative	NA	20	Participants were recruited from the 15 surgical specialty and subspecialty programs at the University of Toronto. Semi-structured interviews and focus groups were conducted with 13 ethics coordinators from the surgical staff and 64 resident trainees. Data were analyzed	Our study used qualitative methodology with focus groups and interviews of postgraduate teachers and trainees to provide an in-depth description of how both groups perceive the pathways to learn ethics in surgical residency.	All coordinators and trainees felt that ethics education was an important component of surgical training. Real cases, varying teaching methods, and teachers with applicable clinical experience were valued. Trainees identified intra-professional and	Ethics education is highly valued by trainees and teachers. Some ethical issues important to trainees are underrepresented in the formal curriculum. Staff surgeons and senior residents are practicing ethicists and role models whose impact on the moral

					qualitatively using modified thematic analysis.		interprofessional conflict, staff behavior perceived to be unethical, and their own lack of experience as challenging issues rarely addressed in the formal ethics curriculum.	development of residents is profound. Their participation in the formal curriculum helps less experienced junior residents realize its value.
Medical students' cases as an empirical basis for teaching clinical ethics	Huijer et al. 2000	Descriptive	NA	NA	The authors analyzed a total of 522 required case reports on ethical dilemmas experienced by interns from September 1995 to May 1999 at the medical school of Vrije Universiteit in Amsterdam. They identified four regularly described and numerous less frequently described topics.	To identify ethical issues that interns encounter in their clinical education and thus build a more empirical basis for the required contents of the clinical ethics curriculum.	The interns addressed a wide range of ethical themes. In 45% of the cases, they mentioned disclosure or non-disclosure of information and informed consent; in 37%, medical decisions at the end of life; in 16%, medical failures; and in 9%, problems transferring patients from one caregiver to another. The interns also identified 27 themes linked to their unique position as interns and 19 themes related to specific types of patients.	Based on self-reported experiences, the authors conclude that clinical ethics teachers should reflect on a multitude of dilemmas. Special expertise is required with respect to end-of-life decisions, truth telling, medical failures, and transferring patients from one caregiver to another. The clinical ethics curriculum should encourage students to voice their opinions and deal with values, responsibilities, and the uncertainty and failings of medical interventions.
Results of a multisite survey of U.S. psychiatry residents on education in professionalism and ethics	Jain et al. 2011	Quantitative	15.5	NA	A written survey was sent to psychiatry residents at seven U.S. residency programs in Spring 2005. The survey was based on an instrument originally developed at the University of New Mexico, consisting of 149 questions in 10 content domains, with 6 questions regarding ethics experiences during training and 5 demographic questions.	The authors assess the perspectives of psychiatry residents about the goals of receiving education in professionalism and ethics, how topics should be taught, and on what ethical principles the curriculum should be based.	A total of 151 psychiatry residents (61%) returned usable responses to our survey. Residents reported receiving a moderate amount of ethics training during medical school (mean: 5.20; scale: 1: None to 9: Very Much) and some ethics training during residency (mean: 4.60). Residents endorsed moderate to moderately strong agreement with all 11 goals of medical education in professionalism and ethics (means: 5.29 to 7.49; scale: 1: Strongly Disagree to 9: Strongly Agree). Respondents were more likely to endorse the value of clinically- and expert-oriented learning methods over web-based educational approaches.	U.S. psychiatry residents endorse a range of goals for education in professionalism and ethics. At the same time, they prefer that these topics be taught in clinically relevant ways and through expert instruction. The value of web-based approaches warrants further investigation.

Pediatricians' reports of their education in ethics	Kesselheim et al. 2008	Quantitative	13	NA	Cross-sectional survey. Two hundred fifty physicians who completed pediatric or medicine/pediatric residency programs in 2004 were randomly selected from the American Medical Association Physician Masterfile. Evaluable responses were received from 150 of 215 eligible pediatricians (70%)	To study pediatricians' assessments of the quality of their ethics education, the impact of various learning methods, and their confidence in confronting ethical dilemmas arising in pediatric practice.	Of 150 respondents, 44.7% rated their ethics education during residency as fair or poor. More than 80% reported that informal discussions with fellow residents and attending physicians had a moderate or major effect on their ethics education, whereas 53.3% reported that formal teaching conferences had a moderate or major impact. Most respondents (60%) reported confidence in addressing 4 of 23 ethical challenges, a moderate pro-portion (40%-60%) reported confidence in addressing 8 of 23 ethical challenges, and fewer (40%) reported confidence in addressing 11 of the ethical challenges. Areas associated with low confidence included ethics in end- of-life care and research ethics.	Efforts are needed to augment formal and informal ethics teaching during residency. Additional studies at both the individual physician and residency program levels are needed to improve the ethics education that pediatricians-in-training receive.
Ethics knowledge of recent paediatric residency graduates: the role of residency ethics curricula	Kesselheim et al. 2016	Quantitative	12	NA	We conducted a cross-sectional survey of recently trained paediatricians which included a validated 23-item instrument called the Test of Residents' Ethics Knowledge for Pediatrics. The sample included paediatricians who completed medical school in 2006– 2008, whose primary specialty was paediatrics or a paediatric subspecialty, and who completed paediatric residency training in 2010–2011. This sample was stratified based on residency programme variables: presence of a formal curriculum in ethics or professionalism, programme size and American Board of Pediatrics certifying exam passage rate. Paediatricians were randomly selected from each stratum for survey participation.	To evaluate the relationship between recently trained paediatricians' ethics knowledge and exposure to a formal ethics or professionalism curriculum during residency.	Among the 370 responding paediatricians (55%), the mean knowledge score was 17.3 (SD 2.2) out of a possible 23. Presence of a formal curriculum in ethics and/or professionalism was not significantly associated with knowledge. Knowledge was lowest on items about parental requests for a child to undergo genetic testing (2 items, 44% and 85% incorrect), preserving patient confidentiality over email (55% incorrect), decision-making regarding life-sustaining technologies (61% incorrect), and decision-making principles such as assent and parental permission (2 items, 47% and 49% incorrect).	This study highlights several areas in which paediatricians' knowledge may be low and that are amenable to targeted educational interventions. These findings should prompt discussion and research among ethicists and educators about how ethics and professionalism curricula can more consistently influence paediatricians' knowledge.
Teaching Ethics in Surgical Training Programs Using a Case-Based	Klingensmith 2008	Descriptive	NA	NA	For the past 5 years, we have used a case-based approach to discuss ethical dilemmas with residents in an interactive conference format. Attendees	To fulfill a curricular need and to address the ACGME competencies of Professionalism and Systems-based practice, we have developed a case-based	Our experiences have shown that a case-based approach to teaching ethics to surgical trainees is feasible, relevant, and important to the education of	In summary, using a case-based approach to teach ethics has been an effective structure to address an often-overlooked aspect of surgical

Format					and participants include medical students, residents, fellows, and surgical attendings, as well as members of the hospital ethics committee, nurses, chaplains, and attendings and trainees from other disciplines. Residents and students collect the cases from their daily experiences and discuss them with the group on a monthly basis. The program was initiated in October 2002 and is ongoing. The format and the participants have evolved some-what over this time period. That evolution and some lessons learned are described in this article.	approach to teaching ethics to trainees at Washington University in Saint Louis.	trainees in the current environment.	curricular planning. Program directors are encouraged to consider this format and to include hospital Ethics committee members, chaplains, social workers, and nurses in the discussions, as we have found this approach to be tremendously rewarding.
Resident-generated versus instructor-generated cases in ethics and professionalism training	Kon 2006	Quantitative	9.5	NA	After two years of course instruction, all second and third year resident (n = 22) were surveyed to assess their perception of the course. These residents had participated in the ethics and professionalism course since their intern year, and had each attended case discussions using both the IGC and RGC format. Surveys were anonymous, and residents were encouraged to add their comments to the survey forms. Residents were asked to score their responses to statements on a five-point Likert scale (strongly disagree to strongly agree). For analysis, we dichotomized responses to each survey question to agree (score of 4 or 5), or not agree (1 through 3).	Therefore, we determined that an assessment of residents' opinions of our course and the case formats presented could serve as a beginning for such inquiry. We therefore chose to focus on resident perceptions in three key areas:<br><br>Do the case discussions cover practical issues that the residents believe they do/will face in clinical practice? Are the case discussions broad enough to cover the wide array of ethical issue they do/will face? And which case format in general do they prefer, which format do they believe facilitates their own learning, and ideally what mix of formats would they prefer? These specific areas were chosen because our education team believed that the differences in case formats would most likely impact these areas. The purpose of this paper is to describe our curriculum and report the findings of our resident survey. These findings may assist other residency programs as they develop ethics and professionalism curricula.	Residents were nearly evenly split between preferring IGC, RGC, or both equally and were split in which they believed best facilitated their learning. In general, however, residents felt that a mix of both formats was ideal regardless of which format they personally preferred (Fig 1). Responses to questions regarding how practical residents found sessions and the breadth of cases covered are presented in Figures 2 and 3. When asked what percentage of cases would ideally be IGCs and RGCs, on average, residents wanted 51% of cases to be resident-generated, and 49% to be instructor-generated.	Based on our relatively small sample at a single institution, we believe that educators should consider incorporating both instructor-generated and resident-generated cases in their ethics and professionalism curricula, and should evaluate the utility of such a model at their own institution.
Ethics and professionalism in the pediatric	Lang et al. 2009	Quantitative	15.5	NA	From February to May 2008, 394 program directors from the Association of Pediatric Program	Since 1982, pediatric residency programs have been asked to evaluate trainees for ethical	Of 386 eligible survey respondents, 233 (60%) returned partial or complete surveys.	Despite requirements to train and evaluate residents in ethics and professionalism,

curriculum: A survey of pediatric program directors					Directors were surveyed.	behavior. In 2007, the Accreditation Council for Graduate Medical Education required documenting teaching and evaluation of professionalism. Pediatric residency pro- gram directors were surveyed to ascertain what they know about the content and process of their ethics and professionalism curricula.	Programs were evenly divided on whether ethics was taught as an organized curriculum or integrated. Professionalism was combined with the ethics curriculum in 27% of pro- grams and taught independently in 38% of programs, but 35% had no professionalism curriculum. More than one third of the respondents did not answer each content and structure question. Approximately two thirds of those who responded stated that their program dedicated 10 hours per year to ethics and professionalism, respectively. Nearly three fourth of programs identified crowding of the curriculum and one third identified lack of faculty expertise as curricular constraints. Respondents expressed interest in more curricular materials from the American Board of Pediatrics or Association of Pediatric Program Directors.	there is a lack of structured curriculum, faculty expertise, and evaluation methodology. Effectiveness of training curricula and evaluation tools need to be assessed if the Accreditation Council for Graduate Medical Education requirements for competencies in these areas are to be meaningfully realized.
Meaning and value in medical school curricula	Lipworth et al. 2012	Qualitative	NA	18	Bioethics and professionalism are standard subjects in medical training programmes, and these curricula reflect particular representations of meaning and practice. It is important that these curricula cohere with the actual concerns of practicing clinicians so that students are prepared for real-world practice. We aimed to identify ethical and professional concerns that do not appear to be adequately addressed in standard curricula by comparing ethics curricula with themes that emerged from a qualitative study of medical practitioners.	Curriculum analysis: Thirty-two prominent ethics and professionalism curricula were identified through a database search and were analysed thematically. Qualitative study: In-depth, semi-structured interviews were conducted with 20 medical practitioners. Participants were invited to reflect upon their perceptions of the ways in which values matter in their practices and their educational	While representations of meaning and value in ethics and professionalism curricula overlap with the preoccupations of practicing clinicians, there are significant aspects of 'real-world' clinical practice that are largely ignored. These fell into two broad domains: (1) 'sociological' concerns about enculturation, bureaucracy, intra-professional relationships, and public perceptions of medicine; and (2) epistemic concerns about making good decisions, balancing different kinds of knowledge, and practising within the bounds of professional protocols.	Our findings support the view that philosophy and sociology should be included in medical school and specialty training curricula. Curricula should be reframed to introduce students to habits of thought that recognize the need for critical reflection on the social processes in which they are embedded, and on the philosophical assumptions that underpin their practice.

The long-term impact of a comprehensive scholarly concentration program in biomedical ethics and medical humanities	Liu et al. 2018	Qualitative	NA	26	There is a strong and growing interest in biomedical ethics and medical humanities (BEMH) within medical education for facilitating key components of medical professionalism and ethics, clinical communication and observational skills, and self-care and reflective practices. This is the first study to examine the impact of a US BEMH SC, from student experience in medical school to post-graduate development, as perceived by graduate physicians.	Graduated students who participated in the BEMH SC or did extensive BEMH research prior to the BEMH SC's establishment (n = 57) were sampled for maximum variation across graduating years. In telephone surveys and interviews, participants discussed the perceived impact of the BEMH SC on (a.) student experience during medical school and (b.) post-graduate development. Interviews were audiotaped, transcribed, and de-identified. The authors iteratively generated a codebook; two raters coded independently, adjudicated codes, and completed inter-rater reliability (IRR) tests. The authors subsequently conducted a team-based thematic analysis, identifying emergent themes.	Nineteen BEMH graduates were interviewed. Results were analyzed according to (a.) student experience and (b.) post-graduate development. Overall, respondents perceived impacts in reinforcing knowledge and skills in clinical ethics; solidifying self-care and reflective practices; refining a sense of professional identity and integrity for ethically challenging situations; and promoting student skills, productivity, and later careers involving BEMH.	The structure and format of a SC may offer additional advantages in promoting student scholarly skill and productivity, career development, and professional identity formation-core competencies identified across clinical training and ethics programs. Our findings indicate that a BEMH SC is effective in achieving a range of desired immediate and post-graduate effects and represent a particularly promising venue for BEMH in medical education.
Ethics curriculum for emergency medicine graduate medical education	Marco et al. 2011	Review	NA	NA	Ethics education is an essential component of graduate medical education in emergency medicine. A sound understanding of principles of bioethics and a rational approach to ethical decision-making are imperative.	In this article, we suggest curricular content, educational approaches, resident evaluation methods, and resources for ethics and professionalism education in emergency medicine.	Ethics curriculum content should include elements suggested by the Liaison Committee on Medical Education, Accreditation Council for Graduate Medical Education, and the Model of the Clinical Practice of Emergency Medicine. Essential ethics content includes ethical principles, the physician-patient relationship, patient autonomy, clinical issues, end-of-life decisions, justice, education in emergency medicine, research ethics, and professionalism.	The appropriate curriculum in ethics education in emergency medicine should include some of the content and educational approaches outlined in this article, although the optimal methods for meeting these educational goals may vary by institution.

Mixed-realism simulation of adverse event disclosure: an educational methodology and assessment instrument	Matos & Raemer 2013	Qualitative	NA	22	Forty-two anesthesiology trainees participated in a 2-part exercise with mixed-realism simulation. The first part took place using a mannequin patient in a simulated operating room where trainees became enmeshed in a clinical episode that led to an adverse event and the second part in a simulated postoperative care unit where the learner is asked to disclose to a standardized patient who systematically moves through epochs of grief response. Two raters scored subjects using an assessment instrument we developed that combines a 4-element behaviorally anchored rating scale (BARS) and a 5-stage objective rating scale.	Thus, the purposes of this study were to (1) demonstrate the feasibility of a structured technique for teaching adverse event disclosure using mixed-realism simulation, (2) develop and begin to validate an instrument for assessing performance, and (3) describe the disclosure practice of a representative cohort of anesthesiology trainees.	The performance scores for elements within the BARS and the 5-stage instrument showed excellent interrater reliability, appropriate range and high internal consistency.	In future work, we can test the learning in subsequent mixed-realism cases to assess learning as compared with groups having other forms of disclosure education. Furthermore, studies of the effectiveness of the learning in a naturalistic environment are possible.
Ethics-in-Oncology Forums.	Mehta et al. 2007	Qualitative	NA	14	We developed an ethics curriculum for hematology/oncology fellows who had already learned medical ethics from medical school and residency programs. The goal of the ethics program was to train fellows in ethics issues specific to hematology/oncology; to raise awareness of ethical issues; and to teach fellows to write, edit, and publish reviews in specific ethical issues	Fellows learned to summarize expert opinions, to understand diversity in cultural concepts relating to ethics, and to crystallize their approaches to ethical dilemmas to selected oncology patients. Fellows were also trained to write ethics discussions in manuscript format, edit the manuscripts, and submit them for publication.	Presentations - Most of the issues related to miscommunications between patient, family, and physicians. Many related to disagreements in treatment, communications in prognosis, or resource allocation. Deliberations - Faculty included hematologists- oncologists, palliative care experts, ethicists, peers, nurses, students, and ethics committee members. Attendance by all the Fellows was required. All of the discussions entailed lively debates among faculty with opposing opinions. One faculty was assigned to facilitate discussions so that that the flow of the discussion could continue despite disagreements. Manuscripts - Of the cases, 12 were written and edited for publication; of these, 6 were published, 1 is in press, 3 were submitted, and 2 are in preparation. Many others were started but not completed. Reasons for noncompletion relate primarily to the fellow losing interest as they progress in the program or after they leave the program or for other	Methods that included readings with discussions appeared to be more beneficial than those in which read- ing material alone was reviewed.23 Some methods are innovative but may not be easy to duplicate at all centers. These included dramatic representations of medical moral dilemmas, film festivals with medical moral dilemmas embedded into the content,15 debates, or games such as “scruples.” Fellows are required to integrate the experience through reviewing it in different phases. Thus, the steps of (a) detecting and selecting the ethical dilemma to present, (b) preparing presentation, (c) reviewing literature, (d) leading discussion about the case, (e) summarizing case, (f) preparing it in manuscript format, (g) editing the manuscript, and (h) submitting the manuscript allows the fellow to review it over and over in different formats toward a desirable end- point, publication.

							unidentified reasons.	
An ethics curriculum for the pediatric residency program: Experience of a university hospital	Moreno et al. 2003	Descriptive	NA	NA	Our program was designed to overcome the following obstacles: (1) time constraints of faculty and residents, (2) scheduling difficulties and lack of continuity, (3) attitudes of residents toward the material, and (4) inadequate ethics training among faculty. In addition to traditional topics in medical ethics, the curriculum focuses on issues that confront residents primarily during their training, issues that may shape their professional values in important ways.	To describe the successful implementation of a structured ethics curriculum for pediatric residents.	This ethics curriculum has been successfully implemented in our own program and offers solutions to common barriers faced by those seeking to implement an ethics curriculum for pediatric residents.	We present the ethics curriculum currently in use at our institution as a tool that may be adopted as it stands or as altered by others as they develop their own program's ethics curriculum. We believe the pro- posed curriculum directly confronts many of the barriers to successful ethics education of pediatric residents.
Incorporating ethics education into the radiology residency curriculum: A model	Oljeski, et al. 2004	Qualitative	NA	16	We conducted an e-mail survey to learn how radiology residency programs responded to the mandate to include the teaching of ethics. Our purpose was twofold: to get a sense of the current state of ethics education in radiology residency programs and to learn possible effective strategies that might be suitable for use in our program to enhance our own curriculum.	With the rapid and continuous introduction of new technologies and techniques in diagnostic and interventional radiology, the radiology resident must master an ever-expanding knowledge base during the 4-year residency program. The resident is expected to develop a level of competence, resulting in board certification. However, successful medical practice also depends on competency in nonmedical areas such as cost-containment, medical–legal issues, business acumen, effective communication, professionalism, and ethics.	Radiology residency programs have responded to the ACGME mandate to incorporate the teaching of ethics in a variety of ways. However, a survey that we conducted indicated that 38% of programs still offer no teaching of ethics to their radiology residents. The ACR videotape about ethics is used by many programs that do incorporate ethics in the curriculum.	On the basis of the enthusiastic discussions at the sessions, the personal viewpoints offered, and the opportunity for residents to listen to the diversity of staff opinions regarding ethics issues, we believe that we achieved our goal of increasing the ethics component of our radiology residency program. Although our method of incorporating ethics teaching into our residency program may not be suitable for other programs, it may serve as a model on which others can expand.
Ethics workshops- Are they effective in improving the competencies of faculty and	Ramalingam et al. 2014	Mixed	13	NA	Responsible conduct of research requires a good knowledge about research ethics. With the recent changes in the clinical trial regulations and the proposed	We wanted to measure the effectiveness of a one day program which was organized using didactic lectures and case scenarios on the knowledge, attitude and skills on	Our findings also support this and found that the short training programs are effective. These types of program evaluations are essential in any Institution as it	This re-emphasizes the need for introduction of ethics training during undergraduate course and also the fact that even a short training program

postgraduates?					introduction of ethics in medical curriculum by the Medical Council of India, there is an urgent need to train the medical faculty and postgraduates in research ethics.	ethics among faculty and postgraduates. This was done using a retro pre-questionnaire. We performed a Kolmogorov Smirnov test to measure the normality, Mann Whitney U-test to test the difference in scores between faculty and postgraduates and a Wilcoxin signed rank test to measure the prepost scores.	provides a concrete measure of the effectiveness of the program. This helps in policy making and also for taking corrective actions in future if found necessary.	in research ethics could be effective.
A randomized trial of teaching bioethics to surgical residents	Robb et al. 2004	Quantitative	17	NA	We randomized 31 first- and second-year surgical residents to either a SP-based seminar or a traditional seminar on informed consent. Immediately after the seminars, we evaluated resident performance in patient encounters on informed consent by using an objective structured clinical examination. Their knowledge of informed consent was also evaluated by using a 20-question short-answer written examination immediately after the seminars and then 3 weeks later.	Our objective was to evaluate the effectiveness of a SP-based seminar compared with a traditional seminar for teaching informed consent to surgical residents. We chose this topic because it is an essential skill for surgeons, and we targeted first- and second-year surgical residents because their clinical responsibilities include ensuring that informed consent is obtained for surgical procedures. Our primary outcome was resident performance in an objective structured clinical examination. Our secondary outcome was resident knowledge using a 20-question short-answer written examination.	Twenty-nine residents completed the study; two withdrew because of an emergency. The SP seminar group had lower SP interview scores on the 22 item checklist compared with the traditional seminar group (57% versus 66%; difference 9%; 95% confidence interval [CI], 17% to 1%, P .03). The SP seminar group also had lower knowledge scores on the questionnaire immediately after the seminar (60% versus 73%; difference 13%; 95% CI, 21% to 4%, P .003). The difference in knowledge scores persisted at 3 weeks (41% for the SP group, 59% for the traditional seminar group; difference 18%; 95% CI, 29% to 7%; P .002). A traditional seminar was superior to an SP-based seminar for teaching informed consent to surgical residents.	Our results suggest ways to improve the approach to teaching informed consent to surgical residents. Our seminars and outcome measures focused on the content of the informed consent interview, such as discussion of risks and benefits. There was less emphasis on teaching the communication and interpersonal aspects. SP-based seminars may be more useful for teaching communication and interpersonal performance, whereas traditional seminars may be better for content. Because effective communication and interpersonal skills are essential in the informed consent process, an observed expert SP interview by a local role model might have more impact on improving resident performance in this domain. In our setting, staff surgeons generally conduct the informed consent discussion of the planned surgical procedure in their office before admission. This discussion often occurs in the absence of the residents because of their responsibilities elsewhere. An idealized recreation of the consent interview using one or more SPs in a class setting could help remedy this problem. To have a lasting and informative impact, postgraduate bioethics

								teaching should ideally involve respected clinicians.
Ethics Education in Neonatal-Perinatal Medicine in the United States	Salih & Boyle 2009	Qualitative	NA	13	We briefly explore the current published data on ethics education in pediatric residency and neonatal- perinatal medicine fellowship programs. Then, we discuss the questions an academic educator may face while developing an ethics curriculum in his/her medical institution. Finally, we present the ethics curriculum that we developed in our neonatal-perinatal medicine fellowship program.	Neonatology is one of the specialties that has immensely benefited from advances in medical technology in the last few decades. These advances have paralleled the rise of the civil rights movements and wider recognition of individual rights. As a result, ethical decision-making has become more complex, involving patients, parents, members of the health care team, and society in general. This has created a need for formal ethics education in neonatal-perinatal medicine fellowship programs.	Neonatology is one of the specialties that has immensely benefited from advances in medical technology. Infants who previously could not survive for medical and surgical reasons now survive. These children sometimes survive with many problems causing parents and patients, physicians and healthcare providers, and society to question the quality of the life saved; even feeling that they are held hostage by technology.43 Physicians spend long hours with parents who request “futile” treatments for their infants, while the physician feels the parent is not acting in the best interest of the infant. The moral dilemmas that neonatologists address in their daily practice are ever increasing.	it becomes essential for the neonatal-perinatal medicine fellowship programs to educate fellows so that they will have the ability to approach ethical dilemmas systematically; a skill that must be learned and cultivated over time.40 This will only improve the most effective paradigm for decision-making in medicine; the shared decision-making model.
Teaching pediatrics residents how to obtain informed consent	Sherman et al. 2005	Quantitative	11.5	NA	All 27 first-year residents in the Northwestern University McGaw Medical Center’s pediatrics residency program participated in a randomized controlled trial with a wait-list control group.14 The study was approved by Institutional Review Boards at Children’s Memorial Institute for Research and at the Northwestern University Feinberg School of Medicine. Each resident provided written informed consent before the study started. The 27 residents were randomly assigned to either the intervention (n 14) or wait-list control (WLC) group (n 13). Quantitative measures were taken after randomization for baseline (pretest) data and again as outcomes (posttest) after the intervention group received the educational session. The intervention group participated in the educational session in addition to performing their usual clinical duties. The WLC group	The purpose of this study was to determine if including a defined educational session on informed consent with specific learning objectives and rigorous outcome measures within a mandatory conference series affects first-year pediatrics residents’ knowledge and attitudes about informed consent.	The quantitative analyses demonstrated that the intervention yielded statistically significant improvements in the measured outcomes. The qualitative analyses confirm the quantitative findings.	A formal session on informed consent in the pediatrics residency educational program positively affects residents’ knowledge and attitudes about informed consent.

					only performed their usual clinical duties. Following the second quantitative outcome measure, the WLC group crossed over and received the educational session while the intervention group returned to their usual clinical duties. Ten participants were then randomly selected from both the intervention (n 5) and the WLC (n 5) groups to undergo a qualitative interview			
Marriage of professional and technical tasks: a strategy to improve obtaining informed consent	Steinemann et al. 2006	Mixed	9.5	16	Surgical trainees were randomized to receive a lecture on obtaining informed consent. Knowledge of, confidence in, and frequency of obtaining consent were assessed. Subsequent first-year residents received combined technical and consent training using a stepwise approach. Residents listed key steps for procedures before and after training, were observed for consent and technical competency on patients, and were assessed on frequency of obtaining consent by follow-up chart review.	Surgical trainees were randomized to receive a lecture on obtaining informed consent. Knowledge of, confidence in, and frequency of obtaining consent were assessed. Subsequent first-year residents received combined technical and consent training using a stepwise approach. Residents listed key steps for procedures before and after training, were observed for consent and technical competency on patients, and were assessed on frequency of obtaining consent by follow-up chart review.	Knowledge and confidence improved after lecture instruction, but consent rate (21%) did not. Stepwise training increased resident awareness of obtaining informed consent as a key step (19% to 77%) and increased frequency of obtaining consent (89% for proctored procedures and 79% in follow-up).	Education alone improves knowledge but not practice of obtaining consent. Teaching the consent process concomitantly with technical training may increase awareness and performance of obtaining informed consent for bedside procedures.
Introducing a curriculum in ethics and professionalism for dermatology residencies	Stoff et al. 2017	Neither	NA	NA	To cover the 15 essential topics in a 3-yearresidency cycle, the subcommittee recommends sessions of approximately 60 minutes' duration every other month. Once the essential topics have been covered, the program may choose from the elective topics. The subcommittee acknowledges that ethical issues may arise within an individual program that may take priority over some of the topics, even core topics, in the model curriculum. The flexible curriculum format lends itself to ad hoc use in analyzing these topics of interest as well.	Ultimately, the goal of this curriculum is to facilitate recognition of ethical issues, foster knowledge about and interest in ethics and ethical reasoning, provide practical tools for resolving ethical dilemmas, and promote professional behavior among dermatology residents. It is hoped that residents will utilize the knowledge, skills, and attitudes gained from the curriculum throughout their professional lives.	The subcommittee designed the curriculum to be learner focused, addressing the specific needs of dermatology residents. A central goal of the curriculum, therefore, is to enable dermatology residents to identify, analyze, and resolve ethical dilemmas most likely to develop in their careers as dermatologists. The content of the curriculum is also specialty specific, emphasizing material particularly relevant to dermatologists, such as privacy and confidentiality, and de-emphasizing other less relevant material, such as reproductive and end-of-life ethics.	The subcommittee recommends a case- based model for content delivery in which residents present cases actually encountered during training or hypothetical cases. In this model, cases under consideration are compared with paradigm cases in ethics, with substantial attention paid to the contextual features of the specific case being presented.
Teaching ethics to paediatrics residents: The centrality of the therapeutic alliance	Taylor et al. 2009	Qualitative	NA	20	The study team directly observed paediatrics residents discussing patients with their faculty preceptors (19 half-day sessions, 76 hours) in an out-patient general paediatrics clinic	To describe the everyday ethics-related issues paediatrics residents encounter as they interact with patients. Our ultimate goal is to use this knowledge to enhance current efforts to teach	A total of 247 cases were recorded. Forty-one of the cases were coded as having ethics-related content. A constant comparative method of qualitative data analysis revealed	Medical faculty tasked with teaching ethics to paediatrics residents can utilise the results of this project to better target and enhance their ethics education efforts directed at

					located in an urban academic medical centre. Each interaction between resident and preceptor about a single patient was considered a case for further analysis.	ethics to paediatrics residents.	that residents were most likely to encounter ethical issues when engaged in the following activities: (i) maintaining a therapeutic alliance with the care-giver (e.g. the parent); (ii) prioritising patient or family needs; (iii) adjusting to the power embodied by the role of doctors, and (iv) distinguishing suboptimal care from abuse or neglect. In addition, our findings indicate that it is through their efforts to maintain the therapeutic alliance with the caregivers of their patients that residents engage in and integrate three processes: developing their medical knowledge; adhering to professional norms, and balancing the power inherent in the doctor's role with their responsibility to serve the patient's interests.	residents in the out-patient setting. Future research could further examine and test these findings in other clinical settings (e.g. adult general medicine).
Education Research: A case-based bioethics curriculum for neurology residents	Tolchin et al. 2015	Quantitative	10	NA	We piloted a case-based bioethics curriculum for neurology residents using the frame- work and topics recommended by the AAN, matched to clinical cases drawn from Columbia's neurologic services. Our primary outcome was residents' ability to analyze and manage ethically complex cases as measured on pre-curriculum and post-curriculum multiple-choice quizzes. Secondary outcomes included pre-curriculum and post-curriculum self-assessed comfort in discussing and managing ethically complex cases, as well as attendance at ethics discussion sessions as compared to attendance at other didactic sessions.	The goal of this training is twofold: 1. To supply the knowledge and cognitive skills necessary for analyzing and making ethical decisions in complex clinical environments 2. To promote the specific attitudes and values deemed necessary to the moral development of the health care professional—a process of "professionalization" or "moral enculturation"3,4	Resident performance on quizzes improved from 75.8% to 86.7% (p 5 0.02). Comfort in discussing ethically complex cases improved from 6.4 to 7.4 on a 10-point scale (p 5 0.03). Comfort in managing such cases trended toward improvement but did not reach statistical significance. Attendance was significantly better at ethics discussions (73.5%) than at other didactic sessions (61.7%, p 5 0.04).	This study provides a basis for a randomized controlled trial of the AAN's ethics curriculum, to determine whether our observed benefits are the result of the formal curriculum rather than ordinary clinical experience. These studies would ideally be performed across multiple training programs to assure generalizability of the results. Because our current results suggest that more attention is needed to manage ethically complex cases, we plan to incorporate into the curriculum 3 or 4 additional case simulations focusing on family discussions regarding goals of care, life support, and patients' religious and cultural values. These simulations and the case discussions should be aligned with specific educational milestones in keeping with the latest recommendations of the Outcome Project of the ACGME and the American

								Board of Medical Specialties.
Ethics and professionalism preparation for psychiatrists-in-training: A curricular proposal	Tsao, & Guedet 2010	Qualitative	NA	NA	The Accreditation Council for Graduate Medical Education (ACGME) Program Requirements for Residency Training in Psychiatry state 'didactic curriculum should include … medical ethics as applied to psychiatric practice' (ACGME, 2007, 2008).<br><br>A number of strategies have been suggested to teach ethics. These include modelling, didactic seminars, case-based method, and clinical 'rounds'. Empirical data suggest that resident physicians, especially female trainees and those over thirty years of age, perceive a need for greater attention to instruction regarding ethical and professional issues, especially in the domains of bioethical principles, informed consent, and care of special populations, and prefer clinical and expert-orientated instruction over formal approaches or independent study	This article proposes a curriculum for senior residents and fellows that uses a case-centred seminar to discuss ethical dilemmas, approaches to analysing ethical issues, and to develop a course of action for resolution. Representative ethical issues and the clinical arenas in which these are most likely to arise are suggested.	We propose a curriculum for postgraduate year 3 or 4 residents and fellows that consists of 12 monthly 90-minute seminars. During each session a senior trainee will present a case from a specific clinical setting or sub-specialism of psychiatry that focuses on one or two core ethical or professionalism principles. The trainee will use Roberts' six-step algorithm as a framework for the case presentation (Roberts et al., 1996). That senior trainee will also be responsible for inviting one or two faculty discussants who supervised the care of the particular patient or who is an expert in the relevant psychiatric specialism.	Because the proposed curriculum emphasizes only a dozen or so ethical and professionalism topics, certain other important topics have likely been either de-emphasized or perhaps excluded altogether. Should these or other topics appear to emerge with some consistency, this curriculum should be adaptable enough to incorporate such material. Finally, though this curriculum is contemplated as an upper level course, consideration can be given to inviting all residents and faculty. If sufficient interest exists, the offering could potentially be expanded into a departmental conference.
Learning by doing: Effectively incorporating ethics education into residency training	Vertrees, et al. 2013	Qualitative	NA	16	We developed and instituted a case-based didactic program in clinical medical ethics for internal medicine residents with the following course objectives: (1) to increase understanding of the theoretical, legal, and practical components of clinical ethics; (2) to apply these lessons to the inherent ethical challenges faced in clinical practice.	To develop and evaluate an experiential educational program for physicians.	Residents unanimously agreed or strongly agreed that (1) medical ethics is a valuable component of medical education, (2) the case-based discussion component of the course was useful, and (3) the overall structure and design of the course was effective. The majority (95 %) agreed/strongly agreed that the didactic component of the course was effective.	An experiential case-based didactic program in medical ethics engaged adult learners and facilitated a comprehensive and clinically relevant educational initiative.
Effectiveness of workshop training in basic principles of good clinical practice among the medical teachers - A cross sectional study	Vora & Shah 2011	Cross sectional	12	NA	The main objective of this study was to show the effectiveness of workshop training in basic principles of Good Clinical Practice among the Medical teachers	A total 125 medical teachers of Government Medical College attached with Sir Takhtsinhji Hospital, a tertiary care hospital, Bhavnagar, Gujarat professionals were participated in Good Clinical Practice training workshop. The study was included total twenty	The total twenty questions were analyzed and compare with the standard key of this. Pre training test score of Question no. 2, 5, 7, 16 was 19(15%), 35 (28%), 21 (17%), 14 (11%) and post training score was 70(88%), 95(76%), 64(51%), 75(60%)	Training workshop with interactive sessions among the participants after some lectures followed by Multiple Choice Questions test not only improved but also update the participations knowledge regarding subject.

						questioners based on the most widely accepted international document forming the base for ICH Harmonised Tripartite Guideline for GCP (ICH-GCP E6), which defines in detail the responsibilities and obligations of parties engaged in clinical research. They were submitted given questioners with answers before and after the workshop training. The data was Mean and SD calculated using SPSS software.	respectively. This number as well as percentage could show more improvement in knowledge regarding basic principle of Good Clinical Practice. In our study results shown that there was improve overall knowledge after the given Good Clinical practice training.	
Anesthesiology trainees face ethical, practical, and relational challenges in obtaining informed consent	Waisel et al. 2009	Qualitative	NA	21	The Program to Enhance Relational and Communication Skills–Anesthesia used professional actors to teach communication skills and relational abilities associated with informed consent. Before attending the program, participants wrote about a challenging informed consent experience. Narratives were analyzed by two researchers following the principles of grounded theory. The researchers independently read the narratives and marked key words and phrases to identify reoccurring challenges described by anesthesiologists. Through rereading of the narratives and discussion, the two researchers reached consensus on the challenges that arose and calculated their frequency.	As part of an educational program on obtaining in- formed consent developed by the Institute of Professionalism and Ethical Practice at Children's Hospital Boston, Massachusetts, we had the opportunity to collect and review narratives by anesthesiology trainees on "challenges in informed consent." This study investigated the trainees' perspectives and educational needs through a qualitative analysis of their narratives.	Analysis of the 39 narratives led to the identification of three types of challenges facing anesthesiologists in obtaining informed consent. Ethical challenges included patient wishes not honored, conflict between patient and family wishes and medical judgment, patient decision-making capacity, and upholding professional standards. Practical challenges included the amount of information to provide, communication barriers, and time limitations. Relational challenges included questions about trainee competence, mistrust associated with previous negative experiences, and misunderstandings between physician and patient or family.	The ethical, practical, and relational challenges in obtaining informed consent colored trainees' views of patient care and affected their interactions with patients. Using participant narratives personalizes education and motivates participants. The richness of narratives may help anesthesiologists to appreciate the qualitative aspects of informed consent.

Teaching Professionalism in Orthopaedic Residency: Efficacy of the American Academy of Orthopaedic Surgeons Ethics Modules	Walsh et al. 2018	Quantitative	14	NA	Two cohorts of orthopaedic residents participated: cohort I completed 14 online ethics modules converted from the 14 AAOS ethics scenarios. For each module, we gave a multiple-choice assessment immediately before the module, immediately afterward, and 3 months afterward. Cohort II completed only the 14-module assessments at similar time intervals without any educational content.	We sought to determine whether residents could obtain content understanding and retention when the material was presented as an online, independent-learning module. If successful, we anticipated that these resources would supplement and enhance our professionalism curriculum.	Cohort I demonstrated improvement in 3-month Post module assessment scores in 11 of the 14 modules, 3 of which had statistical differences in baseline scores for cohort I and cohort II. We observed no statistical difference in scores within cohort II on repeat testing.	This study demonstrates that 11 of the 14 AAOS ethics scenarios, converted to online modules, teach ethical concepts to orthopaedic residents. Orthopaedic residency programs may find it valuable to engage their residents in the ethics scenarios created by the AAOS to complement their ethics curriculum.
