# Supplementary material for: Postgraduate ethics training programs: a systematic scoping review
Source: BMC Med Educ. 2021 Jun 9;21:338. doi: 10.1186/s12909-021-02644-5 (PMC8188952; doi:10.1186/s12909-021-02644-5)
Supplement: Supplementary file 3 — Additional file 3. Tabulated Summaries for Assessing of Ethics. [file 12909_2021_2644_MOESM3_ESM.pdf]

Additional File 3: **Tabulated Summaries for Assessing of Ethics**

Title	Author	Type of study	MERSQI	COREQ	Methodology	Purpose of study	Key findings	Proposed solutions
Bioethics curriculum for paediatrics residents: implementation and evaluation	Cohn (2005)	Quantitative	8.5	-	Administer the feedback form after each session to evaluate the residents' opinion about the session. The new research tool was also trialled to evaluate learning and competency.<br><br>Mentored by Dr Steven Joffe from the Dana Farber Cancer Institute, 5 online ethics vignettes were designed. In May 2004, pilot respondents were asked to identify relevant ethical issues raised by the vignettes and to develop and justify a plan to address the ethical dilemmas.<br><br>A scoring sheet was also developed, and 2 independent readers have scored the pilot responses.	To evaluate the bioethics curriculum for paediatrics resident by developing a new instrument to reliably and validly assess learning and competency in ethics	The curriculum in bioethics is evaluated by 2 methods. After each session, residents complete feedback forms. Residents have given high ratings to the curriculum's relevance to a paediatrician's career, its importance to the care of children, and its ability to effectively convey important knowledge. A formal research study is underway, aimed at creating a new research tool that could be used to evaluate the efficacy of the new ethics curriculum. Mentored by Dr Steven Joffe from the Dana) Farber Cancer Institute, I designed 5 online ethics vignettes.<br>To date, no instrument has been developed that will validly and reliably assess learning and competency in ethics. This study hopes to contribute such a tool that may be utilised in future research in ethics education. If successful, this new instrument will then be used in a future cohort study to evaluate the new curriculum in ethics at Children's Hospital Boston in an effort to define and evaluate ethical competency among our paediatricians.	-
Teaching and assessment of ethics and professionalism: a survey of pediatric program directors	Cook et al. (2013)	Quantitative	7.5	-	From May to August 2012, members of the Association of Paediatric Program Directors (APPD) identified as categorical program directors in the APPD database were surveyed regarding ethics and professionalism practices in their programs, including structure of their curricula, methods of trainee assessment, use of nationally available resources, and	The Accreditation Council for Graduate Medical Education requires residency programs to provide instruction in and evaluation of competency in ethics and professionalism.<br><br>We examined current practices and policies in ethics and professionalism in paediatric training programs, utilization of newly available resources on these topics, and recent concerns	The response rate was 61% (122 of 200). Most paediatric programs continue to teach ethics and professionalism in an unstructured manner. Many paediatric program directors are unaware of available ethics and professionalism resources.<br><br>Although most programs lack rigorous evaluation of trainee competency in ethics and professionalism, 30% (35 of 116) of program directors stated they had not allowed a trainee to graduate or sit for an examination because of	-

					policies regarding social media.	about professional behaviour raised by social media.	unethical or unprofessional conduct. Most programs do not have formal policies regarding social media use by trainees, and expectations vary widely.<br><br>Paediatric training programs are slowly adopting the educational mandates for ethics and professionalism instruction. Resources now exist that can facilitate curriculum development in both traditional content areas such as informed consent and privacy as well as newer content areas such as social media use.	
Teaching and assessing ethics in the newborn ICU	Cummings (2016)	Review	-	-	-	This article reviews currently available teaching methods, pedagogy, and resources in medical ethics, professionalism, and communication, as well as assessment strategies and tools, to help medical educators and practicing clinicians ensure trainees achieve and maintain competency.	A “flipped classroom” approach to teaching medical ethics and professionalism in neonatology may be effective, in which students learn course material online via multimedia modules on their own time, then come to class sessions prepared to engage in a variety of educational activities, including in-depth group discussion, enacted role-play, and simulation.<br><br>This model would engage students with various preferred learning styles and enable mastery of both knowledge and practical skills in ethics and professionalism during training when time is scarce.<br>Our group is in the process of piloting such an innovative ethics and professionalism curriculum for neonatal–perinatal fellows, as well as developing and validating 2 assessment tools.<br><br>Outcomes-based research is still needed to determine optimal teaching methods as well as assessment strategies in ethics and professionalism in neonatology to ensure trainees not only achieve competency in these domains, but also to ensure the formation of professional, virtuous clinicians with excellent moral and ethical reasoning	-

							skills for the ultimate benefit of the patients and their families.	
Assessing Ethics Knowledge: Development of a Test of Ethics Knowledge in Neonatology	Cummings et al. (2018)	Quantitative	12	-	We adapted a published test of ethics knowledge for use in neonatology.<br><br>The novel instrument had 46 true/false questions distributed among 7 domains of neonatal ethics. Content and correct answers were derived from published statements and guidelines.<br><br>Voluntary, anonymous test via e-mailed link were administered to 103 participants, including medical students, neonatology fellows, neonatologists, neonatology nurses, and paediatric ethicists.<br><br>After item reduction, we examined psychometric properties of the resulting 36-item test and assessed overall sample performance.<br><br>We conducted pilot testing with neonatologists and paediatric ethicists (who were excluded from the study) to assess for item clarity and content validity.<br><br>Following cognitive interviews with pilot participants, we revised the instrument.	To develop and validate the Test of Ethics Knowledge in Neonatology (TEK-Neo) with good internal Consistency reliability, item performance, and construct validity that reliably assesses interprofessional staff and trainee knowledge of neonatal ethics	The percentage of correctly answered items relating to informed consent ranged from 13% to 96% (Table II).<br><br>These knowledge gaps are largely consistent with perceived low confidence as reported by neonatal perinatal fellows when faced with similar ethical challenges.	Given recent advances in neonatal genetic screening and testing, it is crucial that neonatal providers be competent in this domain.<br><br>We also found that, similar to previous studies, additional education in bioethics yields improved performance on ethics knowledge tests.<br><br>Future educational efforts should target these consistently poorly performing domains.
Ethics in Child and Adolescent Psychiatry Training: What	Dingle and Kolli (2020)	Quantitative	8	-	CAP program directors were sent an e-mail with a link to an anonymous electronic survey.	This article describes survey results describing ethics/professionalism curricula of US child and	Ninety-nine programs responded with 92 completing the majority of the survey. All had instruction during both training years; reading seminars and lectures were the most common teaching	Continuing to work on developing a national system to systematically share strategies and information across educational programs clearly would benefit CAP

and How Are We Teaching?						adolescent psychiatry (CAP) residency programs.<br><br>This project repeated and expanded upon an earlier survey.	formats. The median number of teaching hours was 10. Teaching was considered inadequate by 22%. Confidentiality, child advocacy, and informed consent were the most frequent ethics topics. Communication, patient care during working hours, and conduct at work were the most common professionalism topics. Faculty and resident opinion differed on certain topics. CAPs were preferred educators in 56.5%. External program resources were available to 87% but over 30% used them rarely or never. Faculty evaluations, 360-degree evaluations, and faculty observations were the most common assessment methods; 38% thought trainee assessments need improvement. Programs were classified into more confident and less confident. More confident programs used available ethics resources more frequently (25% vs 8%, p = 0.30) and had more than the median teaching hours (58% vs 35%, p = 0.035).	residency programs, particularly in the area of ethics and professionalism given its difficulties.
Ethics education in surgical residency programs: a review of the literature	Helft et al. (2009)	Review	-	-	Our primary intent was to review the published, peer-reviewed literature concerning ethics education during general surgery residency. Our goal was to summarize the published literature specifically pertaining to and focusing on ethics education and training in postgraduate surgical training programs. We conducted a literature review using Medline and Ovid databases.<br><br>A second conjunctive search was performed this retrieved 22 articles. We also searched the references of all identified papers and obtained all published articles, webcontent,	This article aims review ethics education in surgical residency programs as these curricula may lead to improvements in patient care	For 2) The authors found statistically significant differences among the final cohort of 29 residents in standardized patient interview scores, and in knowledge scores both immediately after the intervention and 3 weeks later. The differences favoured the traditional seminar group over the standardized patient seminar group.<br><br>The authors concluded that a traditional seminar was superior to a standardized patient-based seminar for teaching informed consent to surgical residents.	The few empirical studies conducted in surgical residencies identified many of the same or similar issues as the experimental ethics curriculum, and all the studies we reviewed showed some level of success in the objectives of the curriculum.<br><br>We advocate for the inclusion of an ethics curriculum into general surgical residencies as a vehicle for meeting the professionalism outcome in the ACGME Outcome Project.

					and abstracts that seemed relevant to our topic.			
A longitudinal simulation-based ethical-legal curriculum for otolaryngology residents	Fanous et al. (2017)	Mixed methods	10	14	Otolaryngology residents were recruited to participate in a yearly half-day ethical–legal module, the curriculum of which spanned 4 years.<br>Each module included: three simulated scenarios, small-group multisource feedback, and large-group debriefings. Scenarios involved encounters with standardized patients. Residents' ethical–legal knowledge was assessed pre- and post-module with multiple-choice questions, and ethical reasoning was assessed by a variety of evaluators during the simulated scenario using a locally developed assessment tool. Participants completed an exit survey at the end of each module.	To develop, implement, and evaluate a longitudinal, simulation-based ethics and legal curriculum designed specifically, for otolaryngology residents.	Prepost knowledge test. Legal and ethics knowledge improved significantly between the pre- and posttest, $P < 0.05$ for each module, with the cumulative pretest mean over 3 years being 3.40, compared to a posttest mean of 4.60.	In conclusion, to our knowledge, this is the first study reporting on a longitudinal simulation-based ethics curriculum tailored specifically to OTL–HNS residents using standardized patients. We have demonstrated evidence that this is an effective way to teach ethics and legal knowledge to OTL–HNS residents.<br>We found both objective and subjective improvements in resident skills, with participants responding to the course positively.
The use of nurses to evaluate house officers' humanistic behavior	Kaplan and Centor (1990)	Quantitative	14.5	-	Using a six-item Likert-scale humanistic behaviour rating form, nurses and ward attendings evaluated 76 PGY-1, PGY-2, and PGY-3 house officers over a six-month period. Nurses and attendings voluntarily evaluated house officers on all inpatient units in both university and Veterans Administration teaching hospitals.	1) To determine whether nurse evaluations of humanistic behaviour discriminate between house officers in an internal medicine training program, and<br><br>2) to compare nurse and attending physician evaluations.	We conclude from the study that nurses can discriminate house officers from one another on the basis of humanistic behaviour and that their evaluations differ significantly from those attending physicians.<br><br>It would, however, be premature to recommend that nurses replace attending as evaluators of humanistic qualities. As it is not clear whether nurses provide better estimates than do attending or ones that are simply different or complementary, we recommend that nurses' and attending' evaluations supplement each other, and perhaps in addition to other measures.	-

							At this stage in the development of measures to assess humanistic behaviour, it may be advisable to place too much trust in one measure. Rather, multiple measures may provide a better overall picture than one used in isolation. Based on our results, we believe that nurses can make a valuable contribution to our ability to measure this very important component of physician practice.	
Ethics knowledge of recent paediatric residency graduates: the role of residency ethics curricula	Kesselheim et al. (2016)	Quantitative	11	-	We conducted a cross-sectional survey of recently trained paediatricians which included a validated 23-item instrument called the Test of Residents' Ethics Knowledge for Paediatrics.<br><br>This sample was stratified based on residency programme variables: presence of a formal curriculum in ethics or professionalism, programme size and American Board of Paediatrics certifying exam passage rate. Paediatricians were randomly selected from each stratum for survey participation.	To evaluate the relationship between recently trained paediatricians' ethics knowledge and exposure to a formal ethics or professionalism curriculum during residency.	Among the 370 responding paediatricians (55%), the mean knowledge score was 17.3 (SD 2.2) out of a possible 23. Presence of a formal curriculum in ethics and/or professionalism was not significantly associated with knowledge.<br><br>Knowledge was lowest on items about parental requests for a child to undergo genetic testing (2 items, 44% and 85% incorrect), preserving patient confidentiality over email (55% incorrect), decision-making regarding life-sustaining technologies (61% incorrect), and decision-making principles such as assent and parental permission (2 items, 47% and 49% incorrect).	These findings should prompt discussion and research among ethicists and educators about how ethics and professionalism curricula can more consistently influence paediatricians' knowledge.
Measuring 'virtue' in medicine	Kotzee and Ignatowicz (2016)	Review	-	-	-	In this paper, we survey the empirical study of medical ethics and find that most studies of doctors' ethics are rules- or principles-based and not virtue-based.	Virtue-based approaches to medical ethics and professionalism seem to hold great promise. Virtue ethics holds that the moral character of the doctor is of the greatest importance in assessing whether their practice of medicine is ethical and professional. It follows that assessment of the ethicality and professionalism with which doctors' practice will have to proceed principally by assessment of doctors' own moral character.	Until researchers who take a virtue approach to medical ethics can begin to operationalise and study virtue concepts in medical education and career, it will be hard to turn virtue-insights into teaching interventions or regulation programmes that are demonstrably effective.<br>True measures of doctors' virtue would be capable of testing for respondents' moral sensitivity, emotion and reasoning all at once

							We surveyed the literature and found that most work on assessing ethics and professionalism in medicine is conducted from a perspective that is rationalistic or cognitivist (and is rules- or principles-based) and not virtue-based. We also found that, of those few studies that are virtue-based or that lend themselves to a virtue-interpretation, none yet lives up to the billing of a true virtue-based assessment of doctors' ethics.	and would have to be able to assess the extent to which these factors are in alignment.
Evaluating professionalism in emergency medicine: clinical ethical competence	Larkin (1999)	Review	-	-	Most ethics assessments to date are conducted retrospectively by risk managers and attorneys. The few prospective evaluations of trainees have focused on single-researcher observations or student attitude surveys that are fraught with observer and recall biases, respectively.<br><br>More reliable and valid methods of identifying clinical ethical competence are needed. This paper reviews a variety of evaluative tools and suggests a three-level approach to monitoring the ethical knowledge, capacity, and real-time performance of emergency medicine residents.	This paper reviews a variety of evaluative tools and suggests a three-level approach to monitoring the ethical knowledge, capacity, and real-time performance of emergency medicine residents.	The evaluation of professionalism in EM is limited by the paucity of evaluation tools available and the lack of time for implementation into an already overfilled curriculum.<br><br>Measures are needed that seamlessly blend with educational opportunities already in place.	1. MEQs on the annual in-service examination;<br><br>2. ethics OSCE stations during ATLS, ACLS, and PALS courses;<br><br>3. blinded videotape review of real-time patient interactions.
Talking about cases in bioethics: the effect of an intensive course on health care professionals	Malek et al. (2000)	Qualitative	-	18	At the Intensive Bioethics Course, a six-day course held annually at Georgetown University, we administered a questionnaire requiring open-ended responses to vignettes both before and after the course.	Our study was designed to provide some of the pieces missing from the published literature concerning the evaluation of bioethics education.<br><br>Specifically, using qualitative techniques, we investigated the effects of a six-day intensive bioethics course on a group of health care professionals.	Clear differences in pre-test and post-test responses were discernible in three qualitative domains: justification, ranking of elements, and recognition of conflicting elements.<br><br>Respondents justified their proposed resolutions more frequently after the course and did so in more depth and detail than before the course.	In retrospect, some adjustments might have been made to expand our findings. Eg. the inclusion of additional details about the scenarios might have enabled respondents to analyse more thoroughly the issues involved. Developing a way to encourage longer discursive responses through interviews would enrich the results. A follow-up instrument could be designed to analyse long term effects of the course.

Professionals' attitudes after a seclusion reduction program: anything changed?	Mann-Poll et al. (2013)	Quantitative	11.5	-	Professionals working on four acute admission wards filled in the Professional Attitudes Toward Seclusion Questionnaire (PATS-Q) before and after a seclusion reduction program. Changes were analysed by comparing mean scores on the PATS-Q.<br><br>After the program, professionals scored significantly higher on 'ethics' and 'more care'.	The purpose of this study was to determine whether professionals from a mental health institute in the Netherlands changed in their attitudes toward seclusion after implementation of a multifaceted seclusion reduction program.	Significant changes in professional attitudes concerning the ethics of using seclusion and involving issues of more care were observed after a seclusion reduction program.<br><br>Mental health professionals moved partly in the direction of ‘‘transformers’’, indicating an increased willingness to question and change their own seclusion practice.	-
Knowledge, perceptions and practices towards medical ethics among physician residents of University of Alexandria Hospitals, Egypt	Mohamed et al. (2012)	Quantitative	13.5	-	The main strengths of this assessment tool are that it is objective, reasonably efficient and logistically feasible. acceptable for that question.<br><br>The latter functions as a scoring guide for trained assessors. Experience in the construction and use of this form of assessment over the years, together with the pre-marking feedback from the student body has tended to minimise the impact of these types of problems.	This cross-sectional study was conducted to assess knowledge, perceptions, practices towards medical ethics of physician residents at university hospitals. A self-administered structured questionnaire was used for knowledge and perceptions and a checklist for observations of doctor–patient interactions in the outpatient setting.	Only 18.0% of the 128 participating residents had obtained their knowledge from their medical education and 29.9% were dissatisfied with the roles played by the ethics committee. Most of the residents had satisfactory knowledge and 60.2% had satisfactory perceptions regarding ethical issues. The lowest perception score was in the domain of disclosing medical errors.<br><br>Only 48.0% of the residents were compliant with the principles of medical ethics in practice and 52.0% of patients were dissatisfied with their treating physicians. The study identified areas of unsatisfactory knowledge and practices towards ethical issues so as to devise means to sensitize residents to these issues and train them appropriately.	-

Analyzing reflective narratives to assess the ethical reasoning of pediatric residents	Moon M et al. (2013)	Qualitative	-	17	Analysis of written narratives focused on two of our ethics curriculum's goals: 1) To raise sensitivity to ethical issues in everyday clinical practice and 2) to enhance critical reflection on personal and professional values as they affect patient care.<br><br>Content analysis of written reflections was guided by a tool developed to identify and assess the level of ethical reasoning in eight domains determined to be important aspects of ethical competence.	This study was conducted to test the concept that content analysis of paediatric residents' personal reflections about ethics experiences can identify changes in ethical sensitivity and reasoning over time.	Based on the assessment of narratives written at two times (12 to 16 months/apart) during their training, residents showed significant progress in two specific domains: use of professional values and use of personal values. Residents did not show decline in ethical reasoning in any domain. This study demonstrates that content analysis of personal narratives may provide a useful method for assessment of developing ethical sensitivity and reasoning.	Our results identify opportunities for further development of a useful method for assessing ethics curricula that employ critical written reflection. Further development of the assessment tool and more extensive application may improve the accuracy of the data and the tool’s capacity to identify changes in ethical reasoning and sensitivity.
Ethics skills laboratory experience for surgery interns	Moon MR et al. (2014)	Quantitative	13	-	We developed curricula and simulated patient cases for 2 core clinical ethics skills--breaking bad news and obtaining informed consent. Educational sessions for each topic included (1) framework development (discussion of interns' current experience, development of a consensus framework for ethical practice, and comparison with established frameworks) and (2) practice with simulated patient followed by peer and faculty feedback. At the beginning and end of each session, we administered a test of confidence and knowledge about the topics to assess the effect of the sessions.	Ethics curricula are nearly universal in residency training programs, but the content and delivery methods are not well described, and there is still a relative paucity of literature evaluating the effect of ethics curricula.<br><br>We detail our development and implementation of a clinical ethics curriculum for surgery interns.	Through faculty-facilitated small group discussion, surgery interns were able to develop frameworks for ethical practice that paralleled established frameworks. Skills-based training in clinical ethics resulted in an increase in knowledge scores and self-reported confidence. Evaluation of 4 annual cohorts of surgery interns demonstrates significant successes and some areas for improvement in this educational intervention.	-
Developing a Knowledge Test for a Neonatal Ethics Teaching Program	Moore et al. (2017)	Quantitative	9.5	-	Using an iterative four-step process, we developed a test for assessing pre- and post-training knowledge of NPM ethics. We first created a	In this study, our aim was to develop a knowledge test to assess the impact of the NPM Ethics Teaching Program on trainees’ knowledge about ethics.	We developed a pre- and post-knowledge test in NPM ethics consisting of 44 multiple choice questions (MCQs), each with five response options. The test takes approximately 60 minutes to	Assessing how trainees use their knowledge when navigating complex and challenging clinical situations; this is a critical learning outcome to measure. We are in the process of developing a

					blueprint of the test, identifying its purpose, length, and format.<br><br>We then weighted the learning outcomes of the NPM Ethics Teaching Program sessions to determine the number of questions that would be asked to assess to each learning outcome. Next, we populated the question bank and constructed a draft test. We obtained feedback from content experts on the draft test and piloted the draft test with former trainees from the NPM Ethics Teaching Program.		complete. It took roughly 15 months to design and pilot the NPM ethics test.<br><br>This test can aid in the assessment of the amount of NPM ethics gained by trainees and contribute to the identification of areas for improvement in teaching and in the overall ethics program. Further iterations of the test will allow for additional assessment of its validity and the efficacy of the teaching program. Given the lack of structured evaluative ethics teaching programs in NPM nationally, this project will act as another step towards the introduction of our NPM Ethics Teaching Program to other Canadian NPM residencies.	communication assessment tool for ethically sensitive scenarios to be used during teaching sessions and at bedside when trainees are directly interacting with parents, to evaluate relationship between knowledge in neonatal ethics and their performance during these situations.
Evaluation of medical ethics competencies in rheumatology: local experience during national accreditation process	Pascual-Ramos et al. (2019)	Quantitative	12.5	-	The station was validated and represented a medical encounter in which the rheumatologist informed a patient with systemic lupus erythematosus with clinically active nephritis about renal biopsy.<br><br>A trained patient–actor and an evaluator were instructed to assess ICP skills (with a focus on kidney biopsy benefits, how the biopsy is done and potential complications) in obtaining formal informed consent, delivering bad news and overall communication with patients. The evaluator used a tailored checklist and form.	Rheumatologists are the primary healthcare professionals responsible for patients with rheumatic diseases and should acquire medical ethical competencies, such as the informed consent process (ICP). The objective clinical structured examination is a valuable tool for assessing clinical competencies. We report the performance of 90 rheumatologist trainees participating in a station designed to evaluate the ICP during the 2018 and 2019 national accreditations.	Candidate performance varied with ICP content and was superior for potential benefit information (achieved by 98.9% of the candidates) but significantly reduced for potential complications (37.8%) and biopsy description (42.2%). Only 17.8% of the candidates mentioned the legal perspective of ICP. Death (as a potential complication) was omitted by the majority of the candidates (93.3%); after the patient–actor challenged candidates, only 57.1% of them gave a clear and positive answer. Evaluators frequently rated candidate communications skills as superior (≥80%), but ≥1 negative aspect was identified in 69% of the candidates.	Ethical competencies are mandatory<br>for professional rheumatologists.<br><br>It seems necessary<br>to include an ethics competency framework in the curriculum throughout the rheumatology residency.

Teaching and evaluation of ethics and professionalism in Canadian family medicine residency programs	Pauls (2012)	Quantitative	12	-	A survey was developed in collaboration with the Committee on Ethics of the College of Family Physicians of Canada.<br><br>The data are reported descriptively and in aggregate.	To document the scope of the teaching and evaluation of ethics and professionalism in Canadian family Medicine postgraduate training programs, and to identify barriers to the teaching and evaluation of ethics and professionalism	By far most Canadian family medicine postgraduate training programs had learning objectives and designated faculty leads in ethics and professionalism, yet there was little curricular time dedicated to these areas and a perceived lack of resident interest and faculty expertise.<br><br>Most programs evaluated ethics and professionalism as part of their end-of-rotation evaluations, but only a small number used novel means of evaluation, and most cited a lack of suitable assessment tools as an important barrier.	-
The ethics objective structured clinical examination	Singer et al. (1993)	Quantitative	11.5	-	Two ten-minute OSCE stations were developed using videotaped encounters between attending physicians and standardized patients.<br><br>The scenarios were evaluated using foreign medical graduates taking an OSCE.<br><br>Each candidate was scored on his or her interaction with a standardized patient in the two OSCE stations by two independent observers.	To develop objective structured clinical examination (OSCE) stations to assess the ability of physicians to address selected clinical-ethical situations, and to evaluate inter-rater agreement in these stations.	The authors produced ethics OSCE stations with face and content validity and satisfactory inter-rater agreement. Ethics OSCE stations may be suitable for evaluating the ability of medical students and residents to address selected clinical- ethical situations.<br><br>OSCE is a promising method to evaluate clinical-ethical abilities of physicians. We have described a method that can be used by others to develop ethics OSCE stations that have face and content validity.<br><br>We have shown that our two ethics OSCE stations possess satisfactory inter-rater agreement to assess specific clinical- ethical abilities in our subject population. We believe that it is premature to recommend widespread use of the ethics OSCE but suggest that medical educators should continue to study its use in the context of educational research.	-
Evaluation of a multicenter ethics objective structured	Singer et al. (1994)	Quantitative	12.5	-	Between January and March 1992, we conducted a	The purpose of this study was to evaluate a six-station ethics objective structured clinical examination (OSCE)	The ethics stations can be used for teaching medical ethics. In addition, the ethics OSCE could be used to evaluate ethics curricula at different medical	Such data may help educators to develop their ethics curricula. For example, an educator at university 1 may decide to enhance ethics

clinical examination					six-station ethics OSCE (as part of a larger OSCE) at three Ontario medical schools.<br><br>The OSCE candidates were a volunteer sample of fourth-year medical students and residents. The ethics stations included two stations on forgoing treatment (intubation and do-not-resuscitate orders), two on confidentiality (HIV and prescription of the birth control pill), and one station each on truth telling and brain death.	on a volunteer sample of 66 medical students and 33 residents from three Ontario medical schools.	schools. Finally, the ethics OSCE could be cautiously used in the evaluation of individual students; although far from perfect, the ethics scores have comparable reliability to the scores for other individual skills in OSCEs currently in use. Moreover, they measure an important clinical skill that at present is not routinely assessed at all.	teaching in decision to forgo treatment, while an educator at university 2 may decide to enhance teaching of confidentiality and truth telling.<br><br>Our stations could be submitted to an interdisciplinary expert panel to enhance the validity of the scoring criteria and set passing scores.
A randomized trial of ethics education for medical house officers	Sulmasy et al. (1993)	Quantitative	16	-	Twenty-five per cent of the house officers received a lecture series (Limited Intervention or LI), 25 per cent received lectures and case conferences, with an ethicist in attendance (Extensive Intervention or EI), and 50 per cent served as controls.<br><br>A post-intervention questionnaire was administered.	We have therefore undertaken a randomized controlled trial of ethics education for medical house officers to determine the impact of such education on house officers' knowledge of medical ethics, confidence in addressing ethical issues, and responses to a simulated case involving resuscitation.	This study provides some initial evidence that a formal ethics education programme for medical house officers can improve the confidence of house officers in addressing ethical problems and can alter their approach to a simulated case. The data indicate that the intervention had more impact upon house officers' confidence and responses to a simulated case than upon knowledge. Many of the knowledge items related to practical concerns (such as whether one could, in an emergency, legally and without a court order transfuse a minor whose parents refused to consent based on their beliefs as Jehovah's Witnesses). Such information ought to be part of the fund of knowledge of all house officers.<br><br>The increase in confidence without a comparable increase in knowledge may seem alarming. The percentage correct on the knowledge test was uniformly low for all groups (C1=53 per cent, C2=58 per cent, LI=56 per cent, EI=57 per cent). There were no significant differences among groups for any particular question nor for summary measures of either the practical	-

							questions (for example, those dealing with informed consent, local law, Jehovah's Witnesses) or the theoretical questions (for example, those dealing with specific philosophical principles or vocabulary, such as the definitions of deontology and utilitarianism).<br><br>The overall confidence scores for the control groups were C1=3 4 and C2=3 8. The confidence scores were 3 9 for the LI group, and 3-8 for the EI group. The confidence score for the aggregate intervention group (LI+EI) was 3 9, which was significantly higher than the total controls (3-6, P<0 05).<br><br>There were no differences between the two control firms or between the total control group and the LI group. However, house officers in the EI group were significantly less likely either to intubate immediately or to elicit patient preferences regarding intubation when compared to all other groups (P<0 05). Forty-three per cent of the EI group elected instead to admit the patient to the floor with 02, antipyretics, and morphine (Table 3).	
Educational intervention is effective in improving knowledge and confidence in surgical ethics-a prospective study	Thirunavukarasu et al. (2010)	Quantitative	8.5	-	General surgery residents at the University of Pittsburgh completed questionnaires measuring attitudes and knowledge about surgical ethics before and after four 60-minute, faculty facilitated seminars implementing the American College of Surgeons ethics curriculum.	We implemented a new curriculum in surgical ethics developed and endorsed by the American College of Surgeons (ACS). The curriculum was taught by a faculty general surgeon with expertise in ethics, and we measured the effectiveness of the curriculum in terms of both knowledge about ethics	The mean score for ethics knowledge for those 28 residents completing the pre-EI questionnaire was 65%. For the 24 residents completing the post-EI questionnaire, the mean score was 71%. For the 21 residents completing both questionnaires, the mean score for ethics knowledge increased from 63% to 70% (P = .013). After the EI, residents reported increased confidence in all 6 core ethical areas. The greatest increase (26%; P = .001) was seen with competition of interests, for which the	Our study demonstrates that residents consider ethics training an important part of the general surgery curriculum and that the ACS text and the seminar style sessions were well received by the participants. In fact, after completing the 4 sessions, more residents thought that ethics training such as described here should be a standard part of residency training.

						and confidence in dealing with ethical problems.	residents had reported the lowest level of confidence before the EI (mean, 3.04). Conversely, the increase in confidence was lowest with confidentiality (10%, P = .011), for which residents had already reported a high level of confidence before the EI (mean, 4.25). This may indicate a ceiling effect.	
Assessing ethical problem solving by reasoning rather than decision making	Tsai et al. (2009)	Mixed	14.5	19	This study used 15 clinical vignettes and the think-aloud method to identify the processes and components involved in ethical problem solving. Subjects included volunteer ethics experts, postgraduate Year 2 residents and pre-clerkship medical students. The interview data were coded using the instruments of the decision score and Ethical Reasoning Inventory (ERI). The ERI assessed the quality of ethical reasoning for a particular case (Part I) and for an individual globally across all the vignettes (Part II)	The purposes of this study were: (i) to create a new instrument to measure doctors' decisions on and reasoning approach towards resolving ethical problems; (ii) to evaluate the scores generated by the new instrument for their reliability and validity, (iii) to compare doctors' ethical reasoning abilities between countries and among medical students, residents and experts	In this study, however, the decision score based on the verbal data could not differentiate among subjects with three different levels of expertise and thus, different reasoning qualities.<br><br>This finding confirms the observations by Beauchamp and Childress that experts cannot be differentiated from novices when judged solely on the correctness of their ethical decision(s). This study thus suggests that the newly developed ERI score for verbal data derived from think-aloud interviews can be used to determine the reasoning quality of people's mental representations.	-
A systems approach to teach core topics across graduate medical education programmes	Varkey and Karlapudi (2008)	Quantitative	8	-	Two subject matter experts in the fields of insurance systems and 1 expert in healthcare QI were invited to conduct institution-wide didactics on the 2 content areas.<br><br>These sessions were held twice for each of the topics in 2007. Each attendee was provided a unique keypad linked to an Audience Response System. This allowed for interactive audience participation and allowed the presenter to receive real-time feedback regarding resident and fellow	Our institution has 86 postgraduate residency and fellowship training programmes serving 1068 learners. Directors of these programmes expressed the need for a centralised approach to teach learners about insurance systems and the basics of QI.	Significant improvement in learner knowledge was noted for all 3 knowledge-based questions for both content areas (P <0.0001). Learner self-assessment of knowledge of insurance systems increased from a pre-session mean of 2.86 to a post-session mean of 3.80 (P <0.0001) and from 3.29 to a post-session mean of 4.17 (P <0.0001) for the QI didactics.	One of the challenges associated with didactic sessions for larger audiences of various specialties include a decreased ability to interact with the speaker.<br><br>An audience response system similar to the one we used within sessions facilitates interactivity between the speaker and the audience, especially in the setting of large number of learners. It also assisted with the assessment of the students and documentation of knowledge competence in the subject matters covered. Although the long-term impact of using audience response systems is not

					understanding of the discussions. It also allowed the speaker(s) to modify the presentation real-time to meet the needs of the audience.<br><br>Participant learners completed 3 pre- and post- session questions assessing learner knowledge of the material as well as 1 question regarding self-assessment of knowledge Responses were elicited using a 5-point Likert scale.			known, other studies suggest that the use of Audience Response System (ARS) allows learners to be more attentive and learn more than in traditional lecture formats.
Supplementing research ethics training in psychiatry residents: A five-tier approach	Viswanath et al. (2018)	Quantitative	7.5	-	We developed a new research ethics training module for psychiatry residents - The Five-Tier Approach. Twenty-five first year psychiatry residents of an academic psychiatric training centre in India participated in this multi-session workshop. Module 1 included the completion of NIH online certification course for research ethics training. Module 2 was a one-hour interactive group discussion on ethical principles in research. Module 3 was a two-hour session consisting of case-based group discussion of nine selected research vignettes. Module 4 involved preparation of an informed consent form. Module 5 was a mock ethics committee role-played by seven students while the larger group observed using a FishBowl technique and provided feedback. Assessments were done during the third and final modules.	Ethics training is a key step in the research supervision of psychiatry trainees and there is need for a structured educational module. In this paper, we have described a new research ethics training module for psychiatry residents – The Five-Tier Approach.	We feel that this five-tier approach is a superior tool for research ethics training in academic institutions, especially in Southeast Asia, where the student-teacher ratios are generally very high.	Formal prepost feedbacks and mid-module assessments could further validate the effectiveness of the program and could help in fine tuning the program to be socio-culturally and academically suited to specific resident groups. Formal prepost feedbacks and mid-module assessments could further validate the effectiveness of the program and could help in fine tuning the program to be socio-culturally and academically suited to specific resident groups

Teaching Professionalism in Orthopaedic Residency: Efficacy of the American Academy of Orthopaedic Surgeons Ethics Modules	Walsh et al. (2018)	Quantitative	12.5	-	Two cohorts of orthopaedic residents participated: cohort I completed 14 online ethics modules converted from the 14 AAOS ethics scenarios. For each module, we gave a multiple-choice assessment immediately before the module, immediately afterward, and 3 months afterward. Cohort II completed only the 14-module assessments at similar time intervals without any educational content.	The purpose of this study was to determine whether the 14 AAOS ethics scenarios, when converted to online modules, provide educational value as demonstrated by module assessment scores indicating content learning and retention.	We have demonstrated the effectiveness of online learning modules to teach ethical principles and to aid retention of this information over a 3-month period.<br><br>Although we empathize with concerns over the lack of an existing validated ethics assessment tool or not creating a control group within cohort I and using traditional lectures, we are encouraged that this successful modality can use public educational resources and does not depend on the availability and interest of an experienced educator. Orthopaedic residency programs who seek to fill limitations in their ethics curriculum may find it helpful to engage their residents in the ethics scenarios created by the AAOS.<br><br>We are encouraged that 11 of the 14 AAOS ethics modules provide a prolonged educational benefit, and as a result, they will become a standard requirement as part of our professionalism curriculum.	-
An assessment of orthopaedic surgeons' knowledge of medical ethics	Wenger and Lieberman (1998)	Quantitative	11	-	102 orthopaedic surgeons completed the survey. Overall, they correctly answered a mean of nineteen (73 per cent) of 26 questions. The respondents appropriately handled questions involving economic aspects, truth telling, confidentiality, and an incompetent colleague.<br><br>However, there was poorer understanding of proper ethical conduct with regard to informed consent, the physician-patient relationship, and end-of-life decision-making.	The purpose of the present study was to evaluate knowledge about ethical issues among attending orthopaedic surgeons and residents in order to assess their ability to handle ethical dilemmas.	This survey demonstrated that orthopaedic surgeons and residents are quite knowledgeable in many critical areas of clinical ethics. Most of the respondents indicated that they would appropriately handle cases involving difficult ethical questions concerning economic issues in clinical orthopaedics, truth-telling, confidentiality, and the approach to an incompetent colleague, and they correctly answered questions testing knowledge. The findings of this survey show that there was greater misunderstanding of proper ethical conduct in the areas of the physician-patient relationship, informed consent, and end-of-life decision-making	Whether educational programs will succeed in remedying these gaps in knowledge must be the subject of future studies

					No significant differences were found, with the numbers available, in overall performance according to site, attending compared with resident status, age, gender, or whether the physician had had training in ethics. Economic, social, and professional forces have increased the medical ethical issues facing orthopaedic surgeons.<br><br>Medical ethics now must be taught in training programs in orthopaedic surgery. Our survey of two orthopaedic surgery training programs demonstrated that orthopaedic surgeons' approach most medical ethical problems appropriately. However, improvement is needed in selected areas.			
A Modified OSCE Assessing the Assimilation and Application of Ethical Principles Relevant to Obstetric and Gynaecological Practice	Woerden et al. (2003)	Quantitative	13	-	26 candidates working in Obstetrics and Gynaecology were presented with 4 questions covering a range of relevant ethical scenarios. Their responses were assessed using a marking schedule. The marking schedule was evaluated against a checklist developed for assessing postgraduate medical examinations. The items in the marking schedule were also assessed to determine the level of agreement between the two examiners. To assess the contribution of each question to the total score, the question to total score correlations were calculated.	To develop and evaluate a modified OSCE assessing the assimilation and application of a range of ethical principles relevant to Obstetric and Gynaecological practice.	This modified OSCE examination demonstrates the feasibility of testing ethical principles relevant to practice in Obstetrics and Gynaecology in candidates for postgraduate posts. It meets most of the criteria laid down in a checklist developed to assess postgraduate medical examinations.	A fully acted out OSCE using actors could have been used, as opposed to a modified OSCE, as it has the advantage of mimicking real life more closely.<br><br>However, it is also much more resource intensive
